# Benchmarking large language models for cell-free RNA diagnostic biomarker discovery

**DOI:** 10.1038/s41467-026-74077-x

**Published:** 2026-06-11

**Authors:** Hunter A. Gaudio, Andrew Bliss, Conor J. Loy, Daniel Eweis-LaBolle, Anne E. Gardella, Iwijn De Vlaminck

**Affiliations:** Meinig School of Biomedical Engineering, Cornell University, Ithaca, NY USA

**Keywords:** Diagnostic markers, Immunological disorders, Machine learning

## Abstract

Large language models can synthesize biomedical knowledge, parse vast amounts of data, and generate code, positioning them as promising tools for biomarker discovery from high-throughput omics data. Here, we benchmark six models from OpenAI, Anthropic, and Google on plasma cell-free RNA datasets spanning three clinical cohorts: Kawasaki disease versus multisystem inflammatory syndrome in children, active tuberculosis versus symptomatic respiratory controls, and myalgic encephalomyelitis/chronic fatigue syndrome versus sedentary controls. We evaluate literature-guided nomination of diagnostic gene panels for downstream machine learning and autonomous construction of end-to-end classifiers from raw count matrices to held-out test predictions. Despite prompt adherence issues, model-nominated panels recapitulate canonical immune pathways and outperform random panels across cohorts, even matching differential gene expression baselines in the tuberculosis cohort. End-to-end automation proves feasible but is model- and task-dependent. One model approaches conventional performance for Kawasaki disease versus multisystem inflammatory syndrome in children, whereas performance decreases for tuberculosis and myalgic encephalomyelitis/chronic fatigue syndrome cohorts. These findings delineate current capabilities and limitations of large language models in diagnostics and open a path for their future use in biomarker discovery.

## Abstract (web summary)

Benchmarking large language models for building diagnostic classifiers from cell-free RNA sequencing data across three cohorts. Models nominate gene panels that recapitulate immune biology. Autonomous classification is model- and task-dependent.

## Introduction

Fewer than one percent of published biomarkers receive US Food and Drug Administration approval, in part because biomarker development remains complex and poorly standardized^1–5^. Therefore, robust, sensitive, and streamlined methods that can integrate existing biological knowledge are needed^6^. Plasma cell-free RNA (cfRNA) profiles offer a rich, minimally invasive readout of tissue injury and disease and show promise across diverse conditions for biomarker discovery^7–18^. Yet, converting cfRNA profiles into clinically actionable signatures remains technically challenging, error-prone, and time-consuming.

Large language models (LLMs) can capture vast biomedical knowledge and can generate executable code, positioning them to automate and potentially improve each step of this process^19,20^. Early successes have been reported in the use of diagnostic LLMs for radiology and pathology^21,22^, and first attempts at LLM-guided omics analysis are emerging^23–26^. Nevertheless, systematic testing in omics-driven biomarker discovery remains limited.

Here, we test whether state-of-the-art LLMs can assist or even outperform purely statistical approaches in developing cfRNA-based diagnostic classifiers. We evaluate LLMs from OpenAI (OpenAI o3 and OpenAI GPT-4o), Anthropic (Claude Opus 4 and Claude 3.7 Sonnet), and Google (Gemini 2.5 Pro and Gemini 2.0 Flash) across three clinical cohorts. First, we compare classifiers built from LLM-nominated gene panels with those built from randomly selected panels and panels selected via differential gene expression (DGE) analysis. Second, we task each model with end-to-end construction of machine learning (ML) classifiers directly from cfRNA gene count data, encompassing feature selection, classifier construction, parameter tuning, and performance reporting. These experiments show where today’s LLMs add value and outline a roadmap for deploying LLMs in diagnostic biomarker development.

## Results

**Fig. 1:**
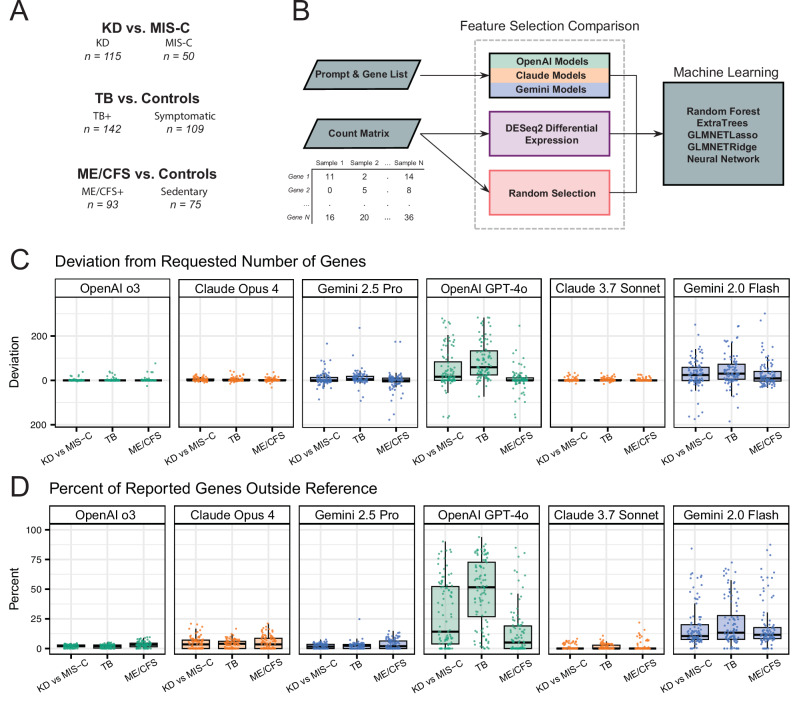
Sample/workflow overview and LLM adherence to prompt requests. **A** Cohort composition and sample sizes for the three clinical datasets analyzed: Kawasaki disease (KD) versus multisystem inflammatory syndrome in children (MIS-C), tuberculosis (TB) versus symptomatic respiratory controls, and myalgic encephalomyelitis/chronic fatigue syndrome (ME/CFS) versus sedentary controls. **B** Feature-selection benchmarking workflow. Gene panels were nominated by three approaches: large language models (OpenAI, Claude, and Gemini), differential gene expression analysis using DESeq2, and random selection. Each panel was then passed to five machine learning classifiers: Random Forest, Extra Trees, GLMNETLasso, GLMNETRidge, and Neural Network. **C** Deviation between the number of genes returned by each LLM and the requested list length (200 genes; long input prompt). **D** Percentage of LLM-reported genes not contained in the cohort reference gene list (long input prompt), summarized across the same 100 attempts as in (**C**). In (**C**, **D**), each point represents one of 100 independent LLM attempts per cohort and model. Box plots show the median (center line), interquartile range (IQR; box), with whiskers extending to 1.5 × IQR.

We assessed six state-of-the-art LLMs (OpenAI o3 and OpenAI GPT-4o, Claude Opus 4 and Claude 3.7 Sonnet, and Gemini 2.5 Pro and Gemini 2.0 Flash) across three clinical cohorts that span a gradient of diagnostic difficulty: Kawasaki disease (KD) (*n* = 115) vs. multisystem inflammatory syndrome in children (MIS-C) (*n* = 50) (mean sequencing depth: 28.1 M reads per sample)^7,27^, tuberculosis (TB) (*n* = 142) vs. symptomatic controls (*n* = 109) (31.7 M reads per sample)^9,28–30^, and myalgic encephalomyelitis/chronic fatigue syndrome (ME/CFS) (*n* = 93) vs. sedentary controls (*n* = 75) (40.8 M reads per sample)^11,31^ (Fig. 1A). KD and MIS-C represent closely related inflammatory conditions with overlapping clinical presentations but distinct pathophysiology: KD is an acute vasculitis of unknown etiology that primarily affects children younger than 5 years, whereas MIS-C is a post-SARS-CoV-2 hyper-inflammatory syndrome that affects older children and shows greater myocardial and gastrointestinal involvement^32–39^. TB presents another diagnostic challenge, with global detection gaps leaving millions of cases undiagnosed annually; the limitations of sputum-based diagnostics and the need to differentiate active TB from other lung diseases make this an important test case for novel biomarker approaches^40–43^. ME/CFS poses the greatest challenge, being defined solely by symptom criteria with no validated biomarkers; rigorous selection of sedentary controls is therefore essential to avoid spurious associations^44–48^.

Together, these cohorts provide a spectrum, ranging from disorders with well-characterized biology to syndromes with no existing molecular tests, that serves as an ideal testing ground for evaluating LLMs. Depending on the task, each LLM received one or more of the following: (i) non-normalized plasma cfRNA read-count matrices (gene × sample), (ii) lists of gene names present in the read-count matrices, and (iii) curated text prompts of varying contextual depth.

### Adherence to input prompts

Before assessing model performance, we evaluated each LLM’s ability to follow the prompt. For every cohort, we asked each LLM 100 times to draw exactly 200 genes from a reference list in order of predictive potential (Fig. 1B). We used either a short or long prompt. The short prompts (Supplementary Information 1, 3 and 5) provided only minimal disease context, whereas the long prompts (Supplementary Information 2, 4 and 6) offered guidance on redundancy reduction and specific approaches for utilizing evidence from the literature (“Methods”). Prompt adherence was quantified as the number of genes returned (Fig. 1C, Supplementary Fig. 1A) and the percentage of selected genes not present in the reference list (Fig. 1D, Supplementary Fig. 1B). Claude 3.7 Sonnet and Claude Opus 4 showed comparable prompt adherence, whereas both OpenAI and Google demonstrated clear improvements in their newest model releases. Notably, Claude 3.7 Sonnet’s prompt adherence was on par with the newer OpenAI and Google models. OpenAI GPT-4o and Gemini 2.0 Flash returned many non-reference features, including protein aliases (e.g., ASCT-1), endogenous retrovirus families (e.g., HERV-W), metabolites (e.g., NADPH), pathway labels (e.g., PI3K), non-human genes (e.g., *SERPINB14*), and even hallucinated features. Adherence did not differ appreciably between short and long prompts, and downstream predictive performance was consistent between prompt types for the KD vs. MIS-C and ME/CFS cohorts (Supplementary Fig. 3). In the TB cohort, Claude Opus 4 showed a significant decrease in performance with longer prompts, while Gemini 2.5 Pro showed a significant increase in performance (Supplementary Table 2); all subsequent analyses used the long-prompt panels for consistency across models and cohorts.

### LLM-nominated gene panels

**Fig. 2:**
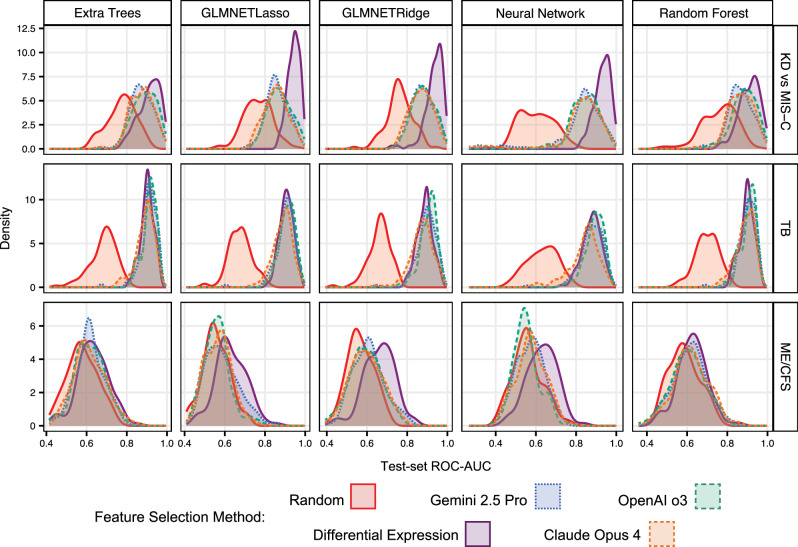
Distribution of held-out test set ROC-AUCs across 100 random train-test splits for gene-panel selection methods and classifiers. Kernel density estimates of receiver operating characteristic area under the curve (ROC-AUC) values computed on the held-out test set for each cohort: Kawasaki disease (KD) versus multisystem inflammatory syndrome in children (MIS-C), tuberculosis (TB), and myalgic encephalomyelitis/chronic fatigue syndrome (ME/CFS) (rows) and each machine learning classifier (columns): Extra Trees, GLMNETLasso, GLMNETRidge, Neural Network, and Random Forest. Curves correspond to feature-selection method: random selection (red, solid), differential expression using DESeq2 (purple, solid), OpenAI o3 (green, dashed), Claude Opus 4 (orange, dashed), and Gemini 2.5 Pro (blue, dashed). Equivalent box-plot distributions for all six LLMs, along with pairwise statistical comparisons between older and newer model generations within each family, are provided in Supplementary Fig. 2, Supplementary Table 1.

To test the predictive value of each gene panel, we used the first 100 genes from each iteration in a machine-learning pipeline (“Methods”). The pipeline trains five classifiers: ridge- and lasso-regularized generalized linear models (GLMNETRidge, GLMNETLasso), random forest, extra trees, and a simple feed-forward neural network. We benchmarked LLM-selected panels against panels based on DGE analysis and randomly selected genes (Fig. 2). Because most of the initial panels selected by the LLMs included non-reference genes, many seeds included fewer than 100 features. Performance declined whenever a seed fell short of 100 usable genes or possessed genes expressed in only a few samples due to a lack of informative candidate features for the models. For the KD vs. MIS-C cohort, across all classifier types, all LLM panels outperformed the random sets but underperformed the DGE panels with no significant differences between the LLMs. For the TB cohort, LLM panel performance matched the DGE panels, even exceeding them in mean receiver operating characteristic area under the curve (ROC-AUC) values in many cases (OpenAI o3 panels outperformed DGE panels across all five ML algorithms, Claude Opus 4 and Gemini 2.5 Pro across three) and outperforming the random sets across all five algorithms. Lastly, for the ME/CFS cohort, every LLM panel trailed the DGE panels and achieved only slight gains over random gene sets (Supplementary Figs. 2, 3). Overall, performance relative to the DGE panels seemed to track the extent of knowledge in the public domain: TB, supported by abundant public data, reached high accuracy; ME/CFS, with a sparse knowledge base, lagged; KD vs. MIS-C fell in between.

### LLMs select disease-relevant genes

**Fig. 3:**
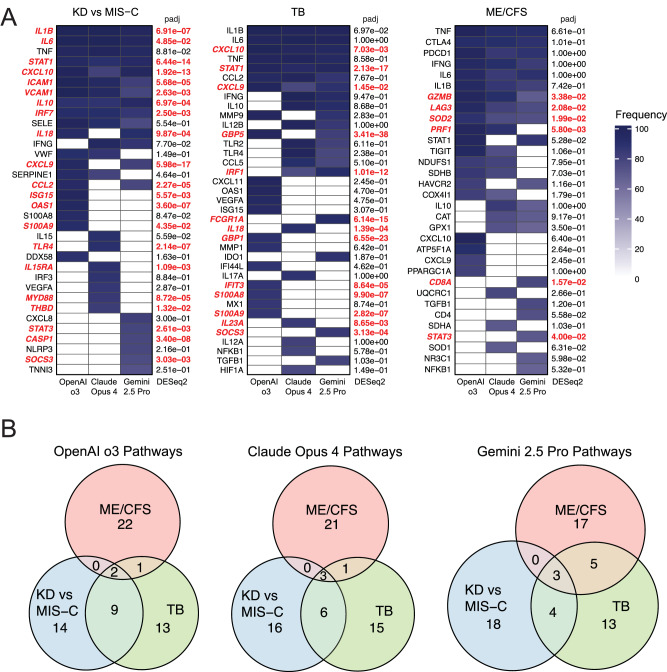
LLM gene selection and biological correlation. **A** Heatmaps summarizing the most frequently selected genes by OpenAI o3, Claude Opus 4, and Gemini 2.5 Pro for each cohort: Kawasaki disease (KD) versus multisystem inflammatory syndrome in children (MIS-C), tuberculosis (TB), and myalgic encephalomyelitis/chronic fatigue syndrome (ME/CFS). Heatmap color intensity indicates gene selection frequency across 100 independent LLM attempts, from 0 (white) to 100 (dark blue). Numerical values at the right of each heatmap are Benjamini–Hochberg-adjusted *P*-values (*p*adj) from DESeq2 differential gene expression analysis of the corresponding cohort; gene symbols in red indicate *p*adj < 0.05. **B** Venn diagrams showing overlap in the top 25 enriched pathways derived from each model’s selected gene panel across the three cohorts. Numbers indicate the count of pathways unique to each cohort or shared across cohorts.

To investigate the biological relevance of the features selected by each model, we first tallied how often each gene appeared across the 100-gene panels and then analyzed the 20 most frequently chosen genes for each clinical cohort (Fig. 3A, Supplementary Fig. 4A). In KD vs. MIS-C, the three LLMs converged strongly: ten genes were selected by all three models, eight of which (*IL1B*, *IL6*, *STAT1*, *CXCL10, ICAM1*, *VCAM1*, *IL10*, *IRF7*) were also significantly differentially expressed in the cfRNA data. TB and ME/CFS selections showed intermediate overlap across LLMs, with three genes selected by all three LLMs and DGE. For TB, OpenAI o3 and Gemini 2.5 Pro selected *GBP5*, a well-validated marker with high statistical significance in the dataset^9^. Across all three top-20 lists (60 total), OpenAI o3 and Gemini 2.5 Pro each captured 26 significantly expressed genes, whereas Claude Opus 4 captured 22. Claude Opus 4 selected the same gene across all three cohorts five times, compared to six times for OpenAI o3 and Gemini 2.5 Pro. Only three features (*IL1B*, *IL6*, and *TNF*) appeared in every LLM’s top 20 list for every cohort, indicating condition specificity.

Using selection frequency as a weight, we performed single-sample gene-set enrichment analysis (ssGSEA) and examined the 25 top-ranked pathways for each condition within each LLM (Fig. 3B, Supplementary Fig. 4B). Each model enriched for immune and inflammatory pathways, such as IL-6, IL-17, and NF-κB signaling, yet the specific pathway mix differed by both LLM and disease (Supplementary Fig. 5). KD vs. MIS-C and TB showed the broadest consensus, whereas ME/CFS displayed the least overlap. This analysis shows that nominated genes were disease-related, and while all LLMs identified biologically plausible features across all cohorts, performance varied between models and with disease complexity, paralleling the trends seen in classifier accuracy.

### LLMs autonomously build classifiers

**Fig. 4:**
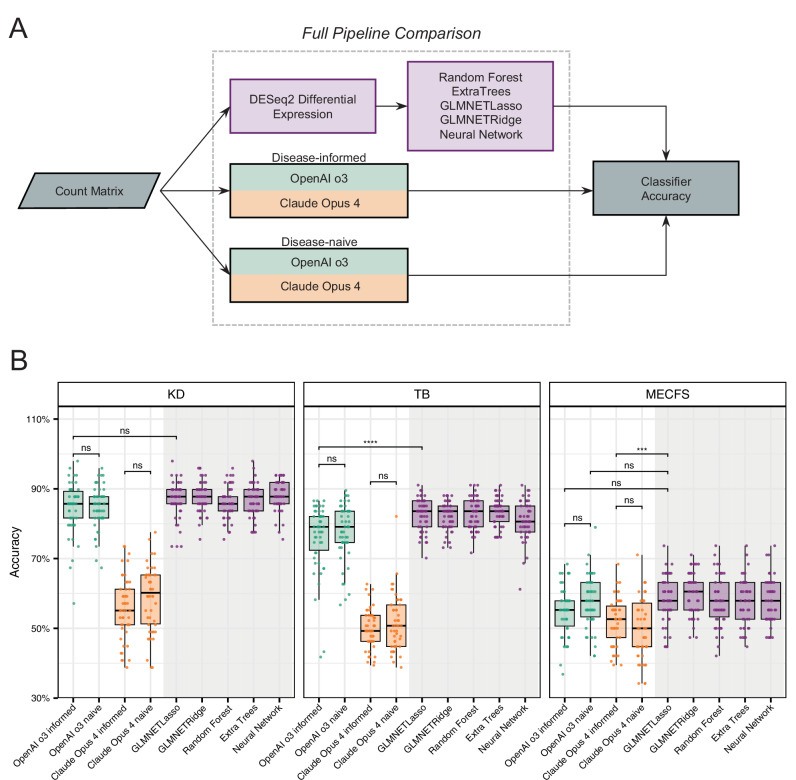
End-to-end modeling performance of LLM-constructed classifiers. **A** Workflow for the full-pipeline comparison. Starting from a plasma cell-free RNA count matrix, classifiers were constructed by three approaches: (i) a standard in-house machine learning pipeline using DESeq2 differential gene expression followed by Random Forest, Extra Trees, GLMNETLasso, GLMNETRidge, and Neural Network classifiers; (ii) disease-informed LLM runs (OpenAI o3 and Claude Opus 4), in which prompts described the disease context and sample type; and (iii) disease-naïve LLM runs (OpenAI o3 and Claude Opus 4), in which prompts omitted any information about condition or sample type. Each approach produced predictions evaluated by classifier accuracy on a held-out test set. **B** Distributions of held-out test accuracy across 50 random train-test splits (seeds) for each cohort: Kawasaki disease (KD) versus multisystem inflammatory syndrome in children (MIS-C), tuberculosis (TB), and myalgic encephalomyelitis/chronic fatigue syndrome (ME/CFS). Each point represents one split. Box colors indicate method: green, OpenAI o3; orange, Claude Opus 4; purple, standard machine learning pipeline classifiers. Gray-shaded regions on the right of each panel indicate standard-pipeline classifiers; unshaded regions indicate LLM-generated classifiers. Box plots show the median (center line), interquartile range (IQR; box), and whiskers extending to 1.5 × IQR. Brackets denote pairwise comparisons; ns indicates not significant, and asterisks indicate significance levels (*P < 0.05, **P < 0.01, ***P < 0.001, ****P < 0.0001). Statistical significance was assessed using two-sided paired *t*-tests (*n* = 50 paired seeds per comparison) with Benjamini–Hochberg correction for multiple comparisons. Exact *P*-values, *t* statistics, and degrees of freedom for all pairwise comparisons are provided in Supplementary Table 3.

After benchmarking gene feature selection by LLMs, we tested whether the LLMs could perform the entire ML workflow autonomously without intervention. Starting with a cfRNA count matrix and corresponding diagnosis labels for the training set, we instructed the LLM to select features and construct and train a binary classifier. We then provided a test set without annotation and instructed the model to classify each sample. Because the LLM returned only discrete class predictions for each sample (without probability estimates or decision scores), score-based metrics such as ROC-AUC and the area under the precision–recall curve (PR-AUC) were not computed. The workflow was executed 50 times with disease-naïve prompts, omitting any information related to condition, cfRNA, sample type, or diagnosis (Supplementary Information 7), and 50 times with disease-informed prompts, which provided details on the condition and sample type and encouraged the LLM to utilize internal or external domain knowledge (Supplementary Information 8–10). In parallel, we performed 50 iterations of the standard ML pipeline using matched random seeds (Fig. 4A).

Among the most advanced models we evaluated (OpenAI o3, Claude Opus 4, Gemini 2.5 Pro), only OpenAI o3 and Claude Opus 4 were capable of consistently performing this task. Gemini 2.5 Pro frequently remained in an indefinite thinking state or terminated with runtime/tool-level errors, preventing consistent completion of both training and inference. For the primary comparisons below, we used GLMNETLasso as the representative standard classifier; full pairwise comparisons against all five standard classifiers are provided in Supplementary Table 3. In the KD vs. MIS-C cohort, OpenAI o3 produced classifiers with mean accuracy approaching that of the GLMNETLasso benchmark (mean = 86.7%) with virtually identical results with disease-informed (84.7%) and disease-naïve (84.8%) prompts (Fig. 4B). Claude Opus 4 performed significantly worse than both OpenAI o3 and the GLMNETLasso benchmark with either prompt type. In the TB cohort, the performance gap widened. The GLMNETLasso benchmark reached 82.6% mean accuracy, whereas OpenAI o3 achieved 76.3% with disease-informed and 77.6% with disease-naïve prompts. Claude Opus 4 again trailed with disease-informed and disease-naïve prompt accuracies of 49.9% and 51.6%, respectively. For the ME/CFS cohort, all methods struggled to classify with high accuracy. Disease-naïve OpenAI o3 runs achieved 57.7% accuracy, statistically indistinguishable from the GLMNETLasso benchmark (57.9%; *P* = 0.966). Disease-informed Claude Opus 4 (52.1%; *P* < 0.001) and disease-naïve Claude Opus 4 (50.0%; *P* < 0.0001) performed significantly worse than the GLMNETLasso benchmark, while disease-informed OpenAI o3 (55.0%) trended lower but did not reach statistical significance after multiple-comparison correction (*P* = 0.066). Across all three clinical cohorts, providing clinical context (disease-informed vs. disease-naïve prompts) did not improve accuracy (KD vs. MIS-C: OpenAI o3 *P* = 0.890, Claude Opus 4 *P* = 0.147; TB: OpenAI o3 *P* = 0.445, Claude Opus 4 *P* = 0.249; ME/CFS: OpenAI o3 *P* = 0.113, Claude Opus 4 *P* = 0.230). Of note, when given disease-informed prompts, both OpenAI o3 and Claude Opus 4 created and used pathway-level variables as features for ML (e.g., “pathway_INTERFERON_GAMMA” and “Pathway_IL1_TNF_score”), but the addition of these features did not improve the performance.

### User experience

For LLM-driven feature selection, when given the prompt and reference gene list, each LLM returned ~200 features within ≈ 2 min. Failures clustered into two categories: (i) refusals citing missing metadata and (ii) sessions that lost the uploaded file. Failures were more frequent with Google models than with OpenAI or Anthropic models. For the end-to-end pipeline task, only OpenAI o3 and Claude Opus 4 could consistently complete both training and inference; Gemini 2.5 Pro timed out or refused execution and was dropped from further analysis. We performed basic reruns for Gemini 2.5 Pro using the same standardized prompt templates and data packaging as the other models; we did not pursue Gemini-specific prompt engineering or alternative data formatting strategies to preserve comparability across LLMs. OpenAI o3 trained in 4–6 min and inferred in 3–5 min with occasional file-access errors. Claude Opus 4 trained more slowly (8–11 min) and failed more often.

## Discussion

Here, we systematically evaluated the capacity of large language models (LLMs) to support liquid-biopsy biomarker discovery and classifier construction from plasma cfRNA-seq data. Across three clinical cohorts spanning a gradient of biological and diagnostic complexity, LLMs consistently recovered biologically meaningful signals despite imperfect prompt adherence. Convergence among models was strongest in the KD^32–37^ vs. MIS-C^34,35,38,39^ and TB^40–43^ cohorts and weakest in ME/CFS^44–48^, a condition with limited mechanistic consensus and comparatively sparse literature. Gene-frequency weighting and pathway enrichment analyses demonstrated that each model converged on canonical immune pathways, interleukin signaling in KD vs. MIS-C, NK-cell and interferon signaling in TB, and mitochondrial stress and inflammatory modules in ME/CFS, indicating that LLM-nominated panels were grounded in disease-relevant biology.

When benchmarked in downstream machine learning pipelines, LLM-selected gene panels consistently outperformed randomly selected panels. In the TB cohort, LLM panels matched, and in several configurations exceeded, those derived from differential gene expression analysis. In contrast, for KD vs. MIS-C and ME/CFS, panels based on differential gene expression retained superior predictive performance. These results suggest that LLM-driven feature nomination can approximate statistically derived panels when disease-relevant signals are well represented in the literature and public data, but may underperform when the discriminative signal is subtle or poorly characterized. Performance differences between model generations illustrate the rapid evolution of LLM capabilities; newer releases from OpenAI and Google showed measurable gains relative to earlier versions, while Claude 3.7 Sonnet demonstrated strong prompt adherence and competitive performance despite not being the newest model evaluated.

A different picture emerged when the LLMs were asked to build complete workflows from raw sequencing counts. OpenAI o3 generated end-to-end classifiers for KD vs. MIS-C that matched the performance of the standard workflow (naïve *P* = 0.138, informed *P* = 0.138) but underperformed the standard workflow in TB (naïve *P* = 2.47 × 10^−5^, informed *P* = 5.30 × 10^−5^) and offered no improvement for ME/CFS (naïve *P* = 0.966, informed *P* = 0.066). Providing additional disease context did not improve accuracy, implying that performance gains stemmed from code-generation rather than domain knowledge. However, because this end-to-end approach returned only discrete class predictions, we could not compute ROC-AUC or PR-AUC, limiting assessment of performance across different decision thresholds, including sensitivity-specificity and precision-recall trade-offs, particularly for imbalanced comparisons. Future benchmarks should require models to output calibrated scores to enable threshold-based analyses.

Several limitations should be considered. First, LLMs did not consistently return exactly the requested number of unique genes, necessitating deterministic truncation to maintain fixed panel sizes. Such variability complicates strict reproducibility and cross-model comparisons. Second, although we explicitly instructed models to avoid using specific prior publications, exposure to relevant literature during pretraining cannot be excluded, and strict verification of compliance is not possible. Third, additional contextual detail in longer prompts did not materially improve adherence or predictive performance, indicating that prompt length alone does not ensure structured outputs. More constrained output schemas or multi-step prompting strategies, such as explicit self-verification passes, may improve reliability. Finally, because end-to-end LLM pipelines produced only class labels, performance comparisons were restricted to accuracy, limiting insight into threshold-dependent behavior, particularly in imbalanced settings.

Hybrid strategies that combine conventional statistical filtering (e.g., sparsity or variance thresholds, DGE prefilters) with LLM-guided literature synthesis may offer a pragmatic path forward, preserving interpretability while leveraging LLMs for candidate prioritization^49–51^. Reproducible deployment will require versioned prompts, explicit model identifiers, and systematic logging of intermediate outputs and failure modes.

Collectively, we find that current LLMs can extract biologically meaningful gene candidates and automate large portions of a bioinformatics pipeline, yet traditional statistical or hybrid workflows still deliver the highest predictive performance in most cases. As LLMs continue to improve, benchmarks like this one will be critical for transparent, reproducible, and clinically safe deployment of language-model diagnostics, shifting the bottleneck from panel discovery to rigorous validation and clinical implementation of AI-generated disease signatures.

## Methods

This research complies with all relevant ethical regulations. This study analyzed de-identified, previously published plasma cfRNA datasets from three clinical cohorts^7,9,11^; no new human samples or data were collected for this work. In each of the original studies, the protocols were approved by the relevant institutional review boards (IRB), and informed consent was obtained from participants (or their parents/guardians) where required by the approving IRB. The approving institutional review boards were: KD vs. MIS-C: University of California San Francisco (UCSF) (#21-33403), Emory University IRB (STUDY00000723), Children’s National Medical Center (Pro00010632), Cornell University (2012010003), and University of California San Diego (#140220); TB: Cornell University (IRB0145569, 1902008555), UCSF (20-32670), the University of Heidelberg Ethics Committee (S-539/2020), the Makerere University School of Medicine Research Ethics Committee (2017-020), the Vietnam National Lung Hospital Ethical Committee (566/2020/NCKH), and the De La Salle Health Sciences Institute Independent Ethics Committee (2020-33-02-A); and ME/CFS: Weill Cornell Medical College (1708018518) and Ithaca College (IRB 1017-12Dx2).

### Sample acquisition and data pre-processing

This study analyzed de-identified plasma cfRNA datasets from three previously published clinical cohorts. Plasma cfRNA data for the Kawasaki disease (KD) and multisystem inflammatory syndrome in children (MIS-C) cohort were obtained from a previously published study of pediatric inflammatory syndromes^7,27^, comprising pediatric patients diagnosed with KD (*n* = 115 used in this study) or MIS-C (*n* = 50 used in this study); full details of patient recruitment, sample collection, sequencing, and quality filtering are described in Loy et al.^7^. Plasma cfRNA data for the tuberculosis (TB) cohort were obtained from a previously published study of plasma cfRNA as a host-response biomarker for tuberculosis^9,28–30^, comprising adults with a cough of at least two weeks’ duration enrolled at outpatient clinics in Uganda, Vietnam, and the Philippines: microbiologically confirmed TB-positive individuals (*n* = 142 used in this study) and symptomatic TB-negative controls (*n* = 109 used in this study); full details are described in Chang, Loy, Eweis-LaBolle, et al.^9^. Plasma cfRNA data for the myalgic encephalomyelitis/chronic fatigue syndrome (ME/CFS) cohort were obtained from a previously published study of cfRNA signatures in ME/CFS^11,31^, comprising ME/CFS cases (*n* = 93 used in this study) and healthy sedentary controls (*n* = 75 used in this study) recruited at three US sites; full details are described in Gardella et al.^11^.

### Sample partitioning

Samples were partitioned into training and test sets at a ratio of 70:30. TB cohort samples were partitioned for ML applications evenly based on collection location and sex. Sample partitioning of the ME/CFS cohort took into consideration collection location, sex, and sequencing batch information.

### In-house machine learning pipeline development

After partitioning and seed-splitting, differential abundance analysis was conducted on the training data. Genes were retained using cohort-specific criteria (KD vs. MIS-C: adjusted *P*-value < 0.05, DESeq2 baseMean > 50, and |log_2_ fold change| > 1; TB: adjusted *P*-value < 0.05 and DESeq2 baseMean > 50; ME/CFS: *P*-value < 0.001 and DESeq2 baseMean > 50). Cohort-specific DGE filtering thresholds were pre-specified to match our previously published analyses of these datasets^7,9,11^ and were not optimized for the present benchmark. Raw counts for the training and test sets were individually normalized with a variance-stabilizing transformation using the DESeq2 package. The test set was transformed using the dispersion function from the training set. Five ML classification algorithms were employed using the R package caret^52^, including generalized linear models with ridge- or lasso-regularization (GLMNETRidge, GLMNETLasso), random forest, extra-trees ensemble, and a feed-forward neural network. Training was performed using fivefold cross-validation and grid search hyperparameter tuning. After training each model, we fixed a decision threshold by maximizing Youden’s J statistic on the training set. We then applied that threshold to the held-out test samples to generate class labels and reported overall classification accuracy as the performance metric.

### Open-domain gene panel selection: LLM input prompt generation

To demonstrate response variability due to input prompt quality, two input prompts were designed for each clinical cohort. The short prompts (Supplementary Information 1, 3 and 5) only stated the two conditions within each cohort, providing no additional insight or guidance on how to approach gene selection. The long prompts (Supplementary Information 2, 4 and 6), however, included information on biological pathway relevance and suggested considerations for handling literature and database evidence, minimizing pathway redundancy, and optimizing diagnostic potential of the selected gene panel. For the KD vs. MIS-C and TB cohorts, the LLMs were instructed to avoid using information from peer-reviewed articles previously published by our lab that used an overlapping dataset and were available online during the time of data generation. Because pretrained LLMs may have internalized such publications during training or indirectly reproduce them via secondary sources, strict verification of compliance is not possible, and results from this arm should be interpreted with this caveat.

### Open-domain gene panel selection: LLM querying

Six proprietary LLMs (OpenAI o3 and OpenAI GPT-4o, Gemini 2.5 Pro and Gemini 2.0 Flash, and Claude Opus 4 and Claude 3.7 Sonnet) were prompted to select genes for a diagnostic panel from a provided reference list. This list, attached in the input prompt as a CSV file, contained all genes from the RNA-seq expression matrices that passed quality control filtering. For each clinical cohort, each input prompt and paired gene list was submitted to each LLM through their respective web interfaces 100 times, generating 100 individual gene panels per prompt type, per LLM, per cohort. This allowed for the assessment of output reproducibility and consistency across models and prompts. The resulting distinct feature sets were then assigned to individual seeds and evaluated in a downstream ML pipeline. The share-memory (conversation-history) feature was turned off; each prompt was submitted in a fresh session, eliminating cross-conversation context.

### Open-domain gene panel selection: input prompt adherence

Each set of LLM-selected gene panels were evaluated for their adherence to the requirements of the input prompt. Specifically, the resulting panel should contain exactly 200 genes, all of which should be found in the provided list. Deviation from 200 genes and the percentage of genes not present on the provided reference list were calculated for each panel.

### Open-domain gene panel selection: gene and enriched pathway analysis

Count matrices of gene selection were generated for each condition and LLM. Using selection frequency as a weight, we performed single-sample gene-set enrichment analysis (ssGSEA) using the GSVA package against Reactome, Gene Ontology biological processes, and Hallmark gene sets, from which we identified the 25 top-ranked pathways shown in Fig. 3B. With the number of LLM selections being used as an analog for gene count, these matrices were also analyzed using QIAGEN Ingenuity Pathway Analysis (IPA).

### Open-domain gene panel selection: performance benchmarking

LLM-selected gene panels were benchmarked against both randomly generated panels and panels derived from DGE analysis using an ML evaluation framework. DGE analysis was performed using the DESeq2 R package^53^ and the cohort-specific filtering criteria described above (Methods: In-house machine learning pipeline development). For each feature set, 100 genes were selected. For LLM-generated features, the LLMs were prompted to return a ranked list of 200 genes (restricted to genes present in the cohort expression matrix), and we deterministically retained the first 100 genes prior to any downstream filtering. This was done because, when prompted for 100 genes, LLM outputs occasionally contained fewer than the requested number of unique genes, which would yield an insufficient feature set for predictive modeling after standard preprocessing (e.g., removal of near-zero variance or extremely sparse features) and make seed-to-seed comparisons inconsistent. Importantly, genes ranked >100 were discarded and not passed through to QC, feature filtering, or model fitting. For each clinical cohort, 100 random seeds were generated, and samples were partitioned into training and testing sets using a 70:30 split, with cohort-specific constraints applied (Methods: Sample partitioning). Feature sets were fed into the in-house ML pipeline, and final model performances were evaluated on the held-out test set using ROC-AUC values. Statistical differences in classifier performance between feature-selection methods were assessed with two-sided Welch’s two-sample *t*-tests, with Benjamini–Hochberg correction for multiple comparisons.

### Predictive pipeline development: LLM input prompt generation

To evaluate the LLM’s capacity to integrate domain knowledge into pipeline development, we generated two prompt types for each clinical cohort: disease-naïve (Supplementary Information 7) and disease-informed (Supplementary Information 8–10). The disease-naïve prompt instructed the LLM to construct a binary classifier without referencing cfRNA, disease context, or related biological concepts. In contrast, the disease-informed prompt explicitly described the input data as plasma-derived cfRNA count matrices and specified the disease phenotypes and control groups for each classification task. The disease-informed prompt encouraged the utilization of both parametric knowledge (encoded during training) and non-parametric knowledge (external retrieval), while explicitly excluding relevant peer-reviewed publications from our research group to prevent data leakage. Because pretrained LLMs may have internalized such publications during training or indirectly reproduce them via secondary sources, strict verification of compliance is not possible, and results from this arm should be interpreted with this caveat. Both prompt configurations specified identical output requirements: (i) notification upon training completion with a request for held-out test data, (ii) generation of predicted class labels for all test samples, and (iii) output of all non-zero weighted features ranked by absolute weight magnitude. Additionally, the LLM was instructed to indicate which features were informed by prior domain knowledge, enabling identification of parametric and non-parametric knowledge contributions to feature selection.

### Predictive pipeline development: LLM querying

Three proprietary LLMs were evaluated for their ability to develop binary classifiers: OpenAI o3, Gemini 2.5 Pro, and Claude Opus 4. Each model was tasked with differentiating disease states within each clinical cohort using either disease-naïve or disease-informed prompts. Only OpenAI o3 and Claude Opus 4 were able to consistently and successfully complete the full pipeline, including model development and prediction generation on held-out test data. The experimental workflow proceeded as follows: the LLM received an input prompt (disease-naïve or disease-informed) through the web interface along with training data in CSV format. Upon completion of binary classifier construction, the LLM requested the provision of held-out test data (CSV format). Training and testing dataset preparation followed the protocols detailed in the Methods: Sample acquisition and data pre-processing and Methods: Sample partitioning sections. Subsequently, the LLM generated two CSV output files as specified in the input prompt: predicted class labels for test samples and ranked non-zero weighted features. These output files were directly downloaded from the interface for downstream analysis. This protocol was executed 50 times per clinical cohort for both prompt conditions (disease-naïve and disease-informed). Each iteration employed unique, randomly generated seeds for training-testing partitioning, with identical seeds maintained across all comparative ML analyses to ensure consistency. The share-memory (conversation-history) feature was turned off; each prompt was submitted in a fresh session, eliminating cross-conversation context.

### Predictive pipeline development: performance benchmarking

Accuracy values for each seed were generated by comparing the LLM-predicted class to the true class. Accuracy values generated by the LLM for each prompt-type were then plotted against accuracy values from the standard pipeline. Differences in classifier performance were assessed with two-sided paired *t*-tests (*n* = 50 paired seeds per comparison). *P*-values were adjusted for multiple comparisons across all pairwise tests within each cohort using the Benjamini–Hochberg procedure. Test statistics (*t*), degrees of freedom, and raw and adjusted *P*-values for all comparisons are reported in Supplementary Table 3.

### Statistics and reproducibility

This study is a computational benchmarking analysis of three previously published plasma cfRNA datasets; no new samples were collected. No statistical method was used to predetermine sample size; sample sizes were fixed by the source cohorts, and no additional exclusions were applied beyond the quality-control criteria of the original studies^7,9,11^. Random train-test splits were used across *n* = 100 independent seeds per condition for feature-selection benchmarking, with comparisons between LLMs assessed using two-sided Welch’s two-sample *t*-tests. End-to-end pipeline benchmarking used *n* = 50 paired seeds per comparison, with identical seeds applied across compared conditions to enable paired *t*-tests. Both analyses used Benjamini-Hochberg correction for multiple comparisons. Analyses were performed in R v4.2.2 using DESeq2 v1.38.0 (differential gene expression), GSVA v1.46.0 (single-sample gene-set enrichment), and the caret package v6.0.94 (machine learning, wrapping glmnet v4.1.8, randomForest v4.7.1.2, extraTrees v1.0.5, and nnet v7.3.19). The investigators were not blinded to allocation during experiments and outcome assessment.

### Reporting summary

Further information on research design is available in the Nature Portfolio Reporting Summary linked to this article.

## Supplementary information

Supplementary Information

Reporting Summary

Transparent Peer Review file

## Data Availability

The raw sequencing data and de-identified RNA-seq count matrices used in this study have been deposited in the NCBI Gene Expression Omnibus under accession codes GSE255555, GSE255071, GSE255073, GSE255074, and GSE293840, with no access restrictions. The pairwise statistical comparisons underlying Fig. 4B are provided in the Supplementary Information (Supplementary Table 3). The processed data used in this study, including the reference gene lists provided to LLMs, the LLM-generated gene panels for each model and cohort, the LLM-generated classifier predictions, and the numerical values underlying all main and Supplementary Figs., are available in the accompanying GitHub repository (https://github.com/adb258/cfrna_ai_manuscript), archived at Zenodo (10.5281/zenodo.19656363)^54^.

## Code availability

All custom code used for data processing, analysis, and figure generation is available on GitHub (https://github.com/adb258/cfrna_ai_manuscript) and archived at Zenodo (10.5281/zenodo.19656363).

## References

[1] Ferreira-Gonzalez, A. et al.Barriers and facilitators to next-generation sequencing use in United States oncology settings: a systematic review. *Future Oncol. Lond. Engl.* **20**, 2765–2777 (2024). 10.1080/14796694.2024.2390821 PMC11572137 39316553

[2] Leclercq, M. et al.Large-scale automatic feature selection for biomarker discovery in high-dimensional OMICs data. *Front. Genet.* **10**, 452 (2019). 31156708 10.3389/fgene.2019.00452 PMC6532608

[3] Li, Y., Mansmann, U., Du, S. & Hornung, R. Benchmark study of feature selection strategies for multi-omics data. *BMC Bioinforma.* **23**, 412 (2022). 10.1186/s12859-022-04962-x PMC9533501 36199022

[4] Benoit, P. et al.Seven-year performance of a clinical metagenomic next-generation sequencing test for diagnosis of central nervous system infections. *Nat. Med.* **30**, 3522–3533 (2024). 39533109 10.1038/s41591-024-03275-1 PMC11645279

[5] Yu, Y., Mai, Y., Zheng, Y. & Shi, L. Assessing and mitigating batch effects in large-scale omics studies. *Genome Biol.* **25**, 254 (2024). 39363244 10.1186/s13059-024-03401-9 PMC11447944

[6] Considine, E. C. The search for clinically useful biomarkers of complex disease: a data analysis perspective. *Metabolites* **9**, 126 (2019). 31269649 10.3390/metabo9070126 PMC6680669

[7] Loy, C. J. et al.Plasma cell-free RNA signatures of inflammatory syndromes in children. *Proc. Natl. Acad. Sci. USA* **121**, e2403897121 (2024). 39240972 10.1073/pnas.2403897121 PMC11406294

[8] Loy, C. J. et al.Nucleic acid biomarkers of immune response and cell and tissue damage in children with COVID-19 and MIS-C. *Cell Rep. Med.* **4**, 101034 (2023). 37279751 10.1016/j.xcrm.2023.101034 PMC10121104

[9] Chang, A. et al.Circulating cell-free RNA in blood as a host response biomarker for the detection of tuberculosis. *Nat. Commun.* **15**, 4949 (2024). 38858368 10.1038/s41467-024-49245-6 PMC11164910

[10] Loy, C., Ahmann, L., De Vlaminck, I. & Gu, W. Liquid biopsy based on cell-free DNA and RNA. *Annu. Rev. Biomed. Eng.* **26**, 169–195 (2024). 38346275 10.1146/annurev-bioeng-110222-111259

[11] Gardella, A. E. et al.Circulating cell-free RNA signatures for the characterization and diagnosis of myalgic encephalomyelitis/chronic fatigue syndrome. *Proc. Natl. Acad. Sci. USA* **122**, e2507345122 (2025). 40789036 10.1073/pnas.2507345122 PMC12377778

[12] Mzava, O. et al.Urine cell-free RNA vs plasma cell-free RNA for monitoring of kidney injury and immune complications. *Clin. Chem.* **71**, 1058–1066 (2025). 40796157 10.1093/clinchem/hvaf082 PMC12490885

[13] Loy, C. J. et al.Molecular characterization of Kawasaki disease subgroups using cell-free RNA profiling. *Sci. Rep.* **15**, 29799 (2025). 40813796 10.1038/s41598-025-15843-7 PMC12354920

[14] Loy, C. et al. Plasma cell-free RNA captures immune dynamics and predicts GVHD after hematopoietic stem cell transplantation. *MedRxiv*10.1101/2024.05.15.24307448 (2025).

[15] Vorperian, S. K. et al.Cell types of origin of the cell-free transcriptome. *Nat. Biotechnol.* **40**, 855–861 (2022). 35132263 10.1038/s41587-021-01188-9 PMC9200634

[16] Moufarrej, M. N. et al.Early prediction of preeclampsia in pregnancy with cell-free RNA. *Nature* **602**, 689–694 (2022). 35140405 10.1038/s41586-022-04410-z PMC8971130

[17] Drag, M. H. & Kilpeläinen, T. O. Cell-free DNA and RNA—measurement and applications in clinical diagnostics with focus on metabolic disorders. *Physiol. Genomics* **53**, 33–46 (2021). 33346689 10.1152/physiolgenomics.00086.2020

[18] Chaddha, M., Rai, H., Gupta, R. & Thakral, D. Integrated analysis of circulating cell-free nucleic acids for cancer genotyping and immune phenotyping of tumor microenvironment. *Front. Genet.* **14**, 1138625 (2023). 37091783 10.3389/fgene.2023.1138625 PMC10117686

[19] Deng, S. et al. The language of cancer: decoding cancer signatures with language models and cfRNA sequencing. *bioRxiv*10.1101/2024.06.29.601341 (2024).

[20] Shen, H., Liu, J., Chen, K. & Li, X. Language model enables end-to-end accurate detection of cancer from cell-free DNA. *Brief. Bioinform.* **25**, bbae053 (2024). 38385880 10.1093/bib/bbae053 PMC10883418

[21] Brown, J. D. et al.Leveraging large language models in radiology research: a comprehensive user guide. *Acad. Radiol.* **32**, 3082–3091 (2025). 39765432 10.1016/j.acra.2024.11.053

[22] Grothey, B. et al.Comprehensive testing of large language models for extraction of structured data in pathology. *Commun. Med.* **5**, 96 (2025). 40164789 10.1038/s43856-025-00808-8 PMC11958830

[23] Awan, A. R., Oveisi, M. & Karimi, M. M. Prompt-based bioinformatics: a new interface for multi-omics analysis. *Nat. Rev. Genet*. 10.1038/s41576-025-00889-0 (2025). 10.1038/s41576-025-00889-0 40789954

[24] Park, N. & Lee, J. H. A systematic multi-LLM AI framework for immunotherapy biomarker discovery and target identification. *bioRxiv*10.1101/2025.06.10.658988 (2025).

[25] Nam, Y. et al.Harnessing artificial intelligence in multimodal omics data integration: paving the path for the next frontier in precision medicine. *Annu. Rev. Biomed. Data Sci.* **7**, 225–250 (2024). 38768397 10.1146/annurev-biodatasci-102523-103801 PMC11972123

[26] Bedi, S. et al.Testing and evaluation of health care applications of large language models: a systematic review. *JAMA* **333**, 319–328 (2025). 39405325 10.1001/jama.2024.21700 PMC11480901

[27] Loy, C., Servellita V, S.-G. A., Bliss, A. & Lenz, J. Series GSE255555 (2024).

[28] Chang, A., Loy, C. & Eweis-LaBolle, D. Series GSE255074 (2024).

[29] Chang, A., Loy, C. & Eweis-LaBolle, D. Series GSE255073 (2024).

[30] Chang, A., Loy, C. & Eweis-LaBolle, D. Series GSE255071 (2024).

[31] Gardella, A. Series GSE293840 (2025).

[32] Burns, J. C. The etiologies of Kawasaki disease. *J. Clin. Invest.* **134**, e176938 (2024). 38426498 10.1172/JCI176938 PMC10904046

[33] Tao, E. & Lang, D. Unraveling the gut: the pivotal role of intestinal mechanisms in Kawasaki disease pathogenesis. *Front. Immunol.* **15**, 1496293 (2024). 39664384 10.3389/fimmu.2024.1496293 PMC11633670

[34] Sharma, C. et al.Multisystem inflammatory syndrome in children and Kawasaki disease: a critical comparison. *Nat. Rev. Rheumatol.* **17**, 731–748 (2021). 34716418 10.1038/s41584-021-00709-9 PMC8554518

[35] Bar-Meir, M. et al.Characterizing the differences between multisystem inflammatory syndrome in children and Kawasaki disease. *Sci. Rep.* **11**, 13840 (2021). 34226639 10.1038/s41598-021-93389-0 PMC8257717

[36] Hara, T. & Sakai, Y. The etiopathogenesis of kawasaki disease: evolving understanding of diverse triggers. *Immun. Inflamm. Dis.* **13**, e70267 (2025). 41017238 10.1002/iid3.70267 PMC12477333

[37] Noval Rivas, M. & Arditi, M. Kawasaki disease vasculitis: from diagnosis to new concepts in pathophysiology and therapeutic approaches. *Annu. Rev. Med.* **77**, 87–102 (2026). 41160750 10.1146/annurev-med-050224-103456

[38] McMurray, J. C., May, J. W., Cunningham, M. W. & Jones, O. Y. Multisystem Inflammatory Syndrome in Children (MIS-C), a post-viral myocarditis and systemic vasculitis—a critical review of its pathogenesis and treatment. *Front. Pediatr.* **8**, 626182 (2020). 33425823 10.3389/fped.2020.626182 PMC7793714

[39] Shyong, O. et al. Multisystem inflammatory syndrome in children: a comprehensive review over the past five years. *J. Intensive Care Med*. 10.1177/08850666251320558 (2025). 10.1177/08850666251320558 40096057

[40] Jeong, J. H., Shim, S. R., Han, S., Hwang, I. & Ihm, C. Diagnostic performance of biomarkers for differentiating active tuberculosis from latent tuberculosis: a systematic review and Bayesian network meta-analysis. *Front. Microbiol.* **15**, 1506127 (2024). 39760075 10.3389/fmicb.2024.1506127 PMC11695403

[41] Pai, M., Dewan, P. K. & Swaminathan, S. Transforming tuberculosis diagnosis. *Nat. Microbiol.* **8**, 756–759 (2023). 37127703 10.1038/s41564-023-01365-3

[42] Chandra, P., Grigsby, S. J. & Philips, J. A. Immune evasion and provocation by Mycobacterium tuberculosis. *Nat. Rev. Microbiol.* **20**, 750–766 (2022). 35879556 10.1038/s41579-022-00763-4 PMC9310001

[43] Scriba, T. J., Maseeme, M., Young, C., Taylor, L. & Leslie, A. J. Immunopathology in human tuberculosis. *Sci. Immunol.* **9**, eado5951 (2024). 39671470 10.1126/sciimmunol.ado5951

[44] Huang, K. et al.Discriminating myalgic encephalomyelitis/chronic fatigue syndrome and comorbid conditions using metabolomics in the UK biobank. *Commun. Med.* **4**, 248 (2024). 39592839 10.1038/s43856-024-00669-7 PMC11599898

[45] Maksoud, R., Magawa, C., Eaton-Fitch, N., Thapaliya, K. & Marshall-Gradisnik, S. Biomarkers for myalgic encephalomyelitis/chronic fatigue syndrome (ME/CFS): a systematic review. *BMC Med.* **21**, 189 (2023). 37226227 10.1186/s12916-023-02893-9 PMC10206551

[46] Nacul, L., Lacerda, E. M., Kingdon, C. C., Curran, H. & Bowman, E. W. How have selection bias and disease misclassification undermined the validity of myalgic encephalomyelitis/chronic fatigue syndrome studies? *J. Health Psychol.* **24**, 1765–1769 (2019). 28810428 10.1177/1359105317695803 PMC5581258

[47] Deumer, U.-S. et al.Myalgic encephalomyelitis/chronic fatigue syndrome (ME/CFS): an overview. *J. Clin. Med.* **10**, 4786 (2021). 34682909 10.3390/jcm10204786 PMC8538807

[48] Arron, H. E. et al.Myalgic encephalomyelitis/chronic fatigue syndrome: the biology of a neglected disease. *Front. Immunol.* **15**, 1386607 (2024). 38887284 10.3389/fimmu.2024.1386607 PMC11180809

[49] Joachimiak, M. P., Caufield, J. H., Harris, N. L., Kim, H. & Mungall, C. J. Gene set summarization using large language models. *arXiv*10.48550/arXiv.2305.13338 (2024).

[50] Toufiq, M. et al.Harnessing large language models (LLMs) for candidate gene prioritization and selection. *J. Transl. Med.* **21**, 728 (2023). 37845713 10.1186/s12967-023-04576-8 PMC10580627

[51] Khan, T. et al.Automating candidate gene prioritization with large language models: from naive scoring to literature-grounded validation. *Bioinformatics* **41**, btaf541 (2025). 41071041 10.1093/bioinformatics/btaf541 PMC12548045

[52] Kuhn, M. Building predictive models in *R* using the caret package. *J. Stat. Softw*. **28**, (2008).

[53] Love, M. I., Huber, W. & Anders, S. Moderated estimation of fold change and dispersion for RNA-seq data with DESeq2. *Genome Biol.* **15**, 550 (2014). 25516281 10.1186/s13059-014-0550-8 PMC4302049

[54] adb258 & Hunter Gaudio. adb258/cfrna_ai_manuscript: Published Repository Release v1.0.0. *Zenodo*10.5281/ZENODO.19656363 (2026).

