# Supplementary material for: Benchmarking large language models for cell-free RNA diagnostic biomarker discovery
Source: Nat Commun. 2026 Jun 11;17:7429. doi: 10.1038/s41467-026-74077-x (PMC13408832; doi:10.1038/s41467-026-74077-x)
Supplement: Supplementary file 1 — Supplementary Information [file 41467_2026_74077_MOESM1_ESM.pdf]

SUPPLEMENTARY INFORMATION TO: Benchmarking large language models for cell-free RNA diagnostic biomarker discovery

**Supplementary Information 1: Open-Domain Gene Panel Selection: KD vs. MIS-C - Short LLM Input Prompt**
You will generate a diagnostic gene panel of 200 genes (listed in order of importance) selected from a list of genes provided in the attached file.<br><br>The panel will differentiate Kawasaki disease (KD) from multisystem inflammatory syndrome in children (MIS-C) using plasma-derived cell-free RNA.<br><br>Exclude any data from the peer-reviewed article titled "*Plasma Cell-free RNA Signatures of Inflammatory Syndromes in Children*".<br><br>Return only the 200 selected genes, listed in descending order of importance.<br><br>Provide the selected genes as a plain text list with one gene per line, separated by single newlines in a code block, without extra spaces or formatting.<br><br>Do not include any extra text, explanations, headers, numbering, or formatting - just the gene list you created.

Supplementary Information 2: Open-Domain Gene Panel Selection: KD vs. MIS-C - Long LLM Input Prompt

You will generate a diagnostic gene panel of 200 genes (listed in order of importance) selected from a list of genes provided in the attached file.

The panel will differentiate Kawasaki disease (KD) from multisystem inflammatory syndrome in children (MIS-C) using plasma-derived cell-free RNA.

Gene Selection Approach:

- Biological Pathway Relevance - Prioritize genes involved in key pathways distinguishing KD from MIS-C:
  - Innate and adaptive immune dysregulation
  - Endothelial dysfunction and vasculitis
  - Hyperinflammation and cytokine storm
  - Cardiac involvement (coronary artery abnormalities, myocarditis)
  - Coagulation abnormalities and thrombo-inflammation
  - Distinct viral vs. bacterial immune response signatures
  - IFN signaling and pattern recognition receptor activation
- Literature and Database Evidence:
  - Use publicly available sources (PubMed, GeneCards, KEGG, Reactome).
  - Exclude any data from the peer-reviewed article titled "*Plasma Cell-free RNA Signatures of Inflammatory Syndromes in Children*".
- Differential Expression Data:
  - Select genes previously reported as differentially expressed between KD and MIS-C.
  - Favor genes with consistent upregulation or downregulation in transcriptomic studies.
- Minimal Redundancy:
  - Ensure diverse pathway representation (limit to 2-3 key regulators per pathway).
  - Select genes with distinct functions rather than multiple redundant markers.
- Diagnostic Potential:
  - Rank genes based on diagnostic value (sensitivity, specificity, robustness in distinguishing KD vs. MIS-C).

Output Instructions:

- Return only the 200 selected genes, listed in descending order of importance.
- Provide the selected genes as a plain text list with one gene per line, separated by single newlines in a code block, without extra spaces or formatting.
- Do not include any extra text, explanations, headers, numbering, or formatting - just the gene list you created.

Supplementary Information 3: Open-Domain Gene Panel Selection: TB vs. Control - Short LLM Input Prompt

You will generate a diagnostic gene panel of 200 genes (listed in order of importance) selected from a list of genes provided in the attached file.

The panel will differentiate tuberculosis (TB)-positive patients from symptomatic TB-negative controls (cough ≥ 2 weeks) using plasma-derived cell-free RNA.

Exclude any data from the peer-reviewed article titled "*Circulating cell-free RNA in blood as a host response biomarker for detection of tuberculosis".*

Return only the 200 selected genes, listed in descending order of importance.

Provide the selected genes as a plain text list with one gene per line, separated by single newlines in a code block, without extra spaces or formatting.

Do not include any extra text, explanations, headers, numbering, or formatting - just the gene list you created.

Supplementary Information 4: Open-Domain Gene Panel Selection: TB vs. Control - Long LLM Input Prompt

You will generate a diagnostic gene panel of 200 genes (listed in order of importance) selected from a list of genes provided in the attached file.

The panel will differentiate tuberculosis (TB)-positive patients from symptomatic TB-negative controls (cough ≥ 2 weeks) using plasma-derived cell-free RNA.

Gene Selection Approach:

- Biological Pathway Relevance - Prioritize genes involved in key TB-related pathways:
  - Immune dysfunction
  - Chronic inflammation
  - Cytokine signaling
  - Granuloma formation and vascular remodeling
  - Host metabolic reprogramming
  - TB-related neuroinflammation and systemic effects
  - TB-induced coagulopathy and cardiac stress
- Literature and Database Evidence:
  - Use publicly available sources (PubMed, GeneCards, KEGG, Reactome).
  - Exclude any data from the peer-reviewed article titled "*Circulating cell-free RNA in blood as a host response biomarker for detection of tuberculosis".*
- Differential Expression Data:
  - Select genes previously reported as differentially expressed between TB-positive patients and symptomatic TB-negative controls.
  - Favor genes with consistent upregulation or downregulation in transcriptomic studies.
- Minimal Redundancy:
  - Ensure diverse pathway representation (limit to 2-3 key regulators per pathway).
  - Select genes with distinct functions rather than multiple redundant markers.
- Diagnostic Potential:
  - Rank genes based on diagnostic value (sensitivity, specificity, robustness in distinguishing TB vs. controls).

Output Instructions:

- Return only the 200 selected genes, listed in descending order of importance.
- Provide the selected genes as a plain text list with one gene per line, separated by single newlines in a code block, without extra spaces or formatting.
- Do not include any extra text, explanations, headers, numbering, or formatting - just the gene list you created.

Supplementary Information 5: Open-Domain Gene Panel Selection: ME/CFS vs. Control - Short LLM Input Prompt

You will generate a diagnostic gene panel of 200 genes (listed in order of importance) selected from a list of genes provided in the attached file.

The panel will differentiate myalgic encephalomyelitis/chronic fatigue syndrome (ME/CFS) from sedentary controls using plasma-derived cell-free RNA.

Return only the 200 selected genes, listed in descending order of importance.

Provide the selected genes as a plain text list with one gene per line, separated by single newlines in a code block, without extra spaces or formatting.

Do not include any extra text, explanations, headers, numbering, or formatting - just the gene list you created.

Supplementary Information 6: Open-Domain Gene Panel Selection: ME/CFS vs. Control - Long LLM Input Prompt

You will generate a diagnostic gene panel of 200 genes (listed in order of importance) selected from a list of genes provided in the attached file.

The panel will differentiate myalgic encephalomyelitis/chronic fatigue syndrome (ME/CFS) from sedentary controls using plasma-derived cell-free RNA.

Gene Selection Approach:

- Biological Pathway Relevance - Prioritize genes involved in key ME/CFS-related pathways:
    - Dysregulated immune response (chronic immune activation, T cell/NK cell dysfunction, T cell exhaustion)
    - Mitochondrial dysfunction and impaired energy metabolism
    - Oxidative stress and reactive oxygen species (ROS) imbalance
    - Neuroinflammation and microglial activation
    - Dysautonomia and vascular abnormalities
    - HPA axis dysregulation and abnormal stress response
    - Altered gut-brain-immune axis signaling
- Literature and Database Evidence:
    - Use publicly available sources (PubMed, GeneCards, KEGG, Reactome).
- Differential Expression Data:
    - Select genes previously reported as differentially expressed between ME/CFS and healthy (but sedentary) controls.
    - Favor genes with consistent upregulation or downregulation in transcriptomic studies.
- Minimal Redundancy:
    - Ensure diverse pathway representation (limit to 2-3 key regulators per pathway).
    - Select genes with distinct functions rather than multiple redundant markers.
- Diagnostic Potential:
    - Rank genes based on diagnostic value (sensitivity, specificity, robustness in distinguishing ME/CFS vs. healthy (but sedentary) controls).

Output Instructions:

- Return only the 200 selected genes, listed in descending order of importance.
- Provide the selected genes as a plain text list with one gene per line, separated by single newlines in a code block, without extra spaces or formatting.
- Do not include any extra text, explanations, headers, numbering, or formatting - just the gene list you created.

Supplementary Information 7: Predictive Pipeline Development: Disease-Naïve LLM Input Prompt

Task:

Please develop the best possible binary classifier that distinguishes between two classes (0 and 1).

Training data:

- seed_*X*_all.counts.train - first row = class labels (0/1); remaining rows = feature counts; sample IDs are in the header row.

Requirements:

- Performance - optimize predictive accuracy. When you're satisfied, freeze the model, tell me you're ready and I'll supply the held-out test set for evaluation.
- Do not retrain the model once the test set (seed_*X*_all.counts.test) is supplied.

Deliverables once the test data is supplied:

- predictions.csv - after the model is frozen and the test set is provided, load it once and output two columns: sample_id,predicted_class (0 or 1).
- features_weights_genes_sorted.csv - columns = feature,weight,knowledge_source (knowledge_source ∈ {data,intrinsic,external}), sorted by |weight| descending.

Do not provide a script so that I can generate the deliverables myself. Generate the deliverables and provide them as a downloadable link.

Feel free to take the time you need - robustness and clarity matter more than speed.

Supplementary Information 8: Predictive Pipeline Development: Disease-Informed (KD vs. MIS-C) LLM Input Prompt

Task:
Please develop the best possible binary classifier that distinguishes Kawasaki disease (KD = 0) from multisystem inflammatory syndrome in children (MIS-C = 1).

Training data:

- seed_*X*_all.counts.train - the plasma-derived cell-free RNA expression matrix; the first row contains the class label for every sample (0 = KD-positive, 1 = MIS-C-positive).

Requirements:

- Leverage domain knowledge – incorporate published KD/MIS-C biology wherever helpful. Examples (use as many as you deem beneficial):
    - Known marker genes
    - Relevant pathways
    - Public KD and MIS-C gene signatures from recent literature
    - Exclude any data from the peer-reviewed article titled "*Plasma Cell-free RNA Signatures of Inflammatory Syndromes in Children*".
- Explain how the prior knowledge is integrated (feature selection, pathway scores, priors in the model, ensemble rules, etc.).
- Performance - still optimize predictive accuracy; justify any trade-off you make between interpretability and raw metrics.
- When you're satisfied, freeze the model, tell me you're ready and I'll supply the held-out test set for evaluation.
- Do not retrain the model once the test set (seed_*X*_all.counts.test) is supplied.

Deliverables once the test data is supplied:

- predictions.csv - after the model is frozen and the test set is provided, load it once and output two columns: sample_id,predicted_class (0 or 1).
- features_weights_genes_sorted.csv - columns = feature,weight,knowledge_source

  (knowledge_source ∈ {data,intrinsic,external}), sorted by |weight| descending.

Do not provide a script so that I can generate the deliverables myself. Generate the deliverables and provide them as a downloadable link.

Feel free to take the time you need - robustness and clarity matter more than speed.

Supplementary Information 9: Predictive Pipeline Development: Disease-Informed (TB vs. Control) LLM Input Prompt

Task:

Please develop the best possible binary classifier that distinguishes tuberculosis (TB)-positive patients from symptomatic TB-negative controls (cough ≥ 2 weeks).

Training data:

- seed_*X*_all.counts.train - the plasma-derived cell-free RNA expression matrix; the first row contains the class label for every sample (0 = symptomatic TB-negative control, 1 = TB-positive).

Requirements:

- Leverage domain knowledge – incorporate published TB biology wherever helpful. Examples (use as many as you deem beneficial):
  - Known marker genes
  - Relevant pathways
  - Public TB gene signatures from recent literature
  - Exclude any data from the peer-reviewed article titled "*Circulating cell-free RNA in blood as a host response biomarker for detection of tuberculosis".*
- Explain how the prior knowledge is integrated (feature selection, pathway scores, priors in the model, ensemble rules, etc.).
- Performance - still optimize predictive accuracy; justify any trade-off you make between interpretability and raw metrics.
- When you're satisfied, freeze the model, tell me you're ready and I'll supply the held-out test set for evaluation.
- Do not retrain the model once the test set (seed_*X*_all.counts.test) is supplied.

Deliverables once the test data is supplied:

- predictions.csv - after the model is frozen and the test set is provided, load it once and output two columns: sample_id,predicted_class (0 or 1).
- features_weights_genes_sorted.csv - columns = feature,weight,knowledge_source (knowledge_source ∈ {data,intrinsic,external}), sorted by |weight| descending.

Do not provide a script so that I can generate the deliverables myself. Generate the deliverables and provide them as a downloadable link.

Feel free to take the time you need - robustness and clarity matter more than speed.

Supplementary Information 10: Predictive Pipeline Development: Disease-Informed (ME/CFS vs. Control) LLM Input Prompt

Task:
Please develop the best possible binary classifier that distinguishes myalgic encephalomyelitis/chronic fatigue syndrome (ME/CFS) from sedentary controls.

Training data:

- seed_*X*_all.counts.train - the plasma-derived cell-free RNA expression matrix; the first row contains the class label for every sample (0 = sedentary control, 1 = ME/CFS-positive).

Requirements:

- Leverage domain knowledge – incorporate published ME/CFS biology wherever helpful. Examples (use as many as you deem beneficial):
    - Known marker genes
    - Relevant pathways
    - Public ME/CFS gene signatures from recent literature
- Explain how the prior knowledge is integrated (feature selection, pathway scores, priors in the model, ensemble rules, etc.).
- Performance - still optimize predictive accuracy; justify any trade-off you make between interpretability and raw metrics.
- When you're satisfied, freeze the model, tell me you're ready and I'll supply the held-out test set for evaluation.
- Do not retrain the model once the test set (seed_*X*_all.counts.test) is supplied.

Deliverables once the test data is supplied:

- predictions.csv - after the model is frozen and the test set is provided, load it once and output two columns: sample_id,predicted_class (0 or 1).
- features_weights_genes_sorted.csv - columns = feature,weight,knowledge_source

  (knowledge_source ∈ {data,intrinsic,external}), sorted by |weight| descending.

Do not provide a script so that I can generate the deliverables myself. Generate the deliverables and provide them as a downloadable link.

Feel free to take the time you need - robustness and clarity matter more than speed.

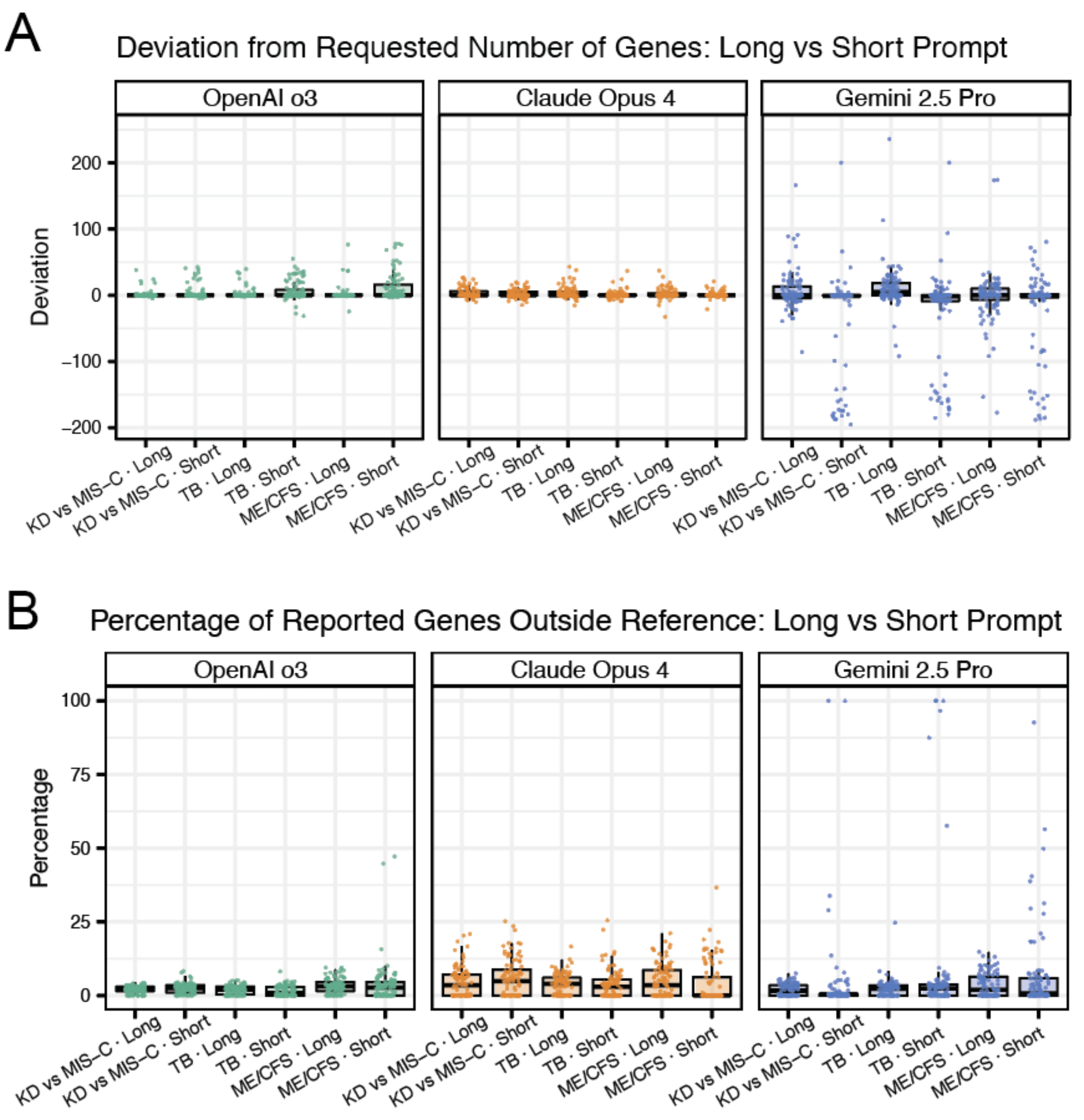

Supplementary Figure 1: Prompt adherence comparison by prompt length. (A) Deviation between the number of genes returned by each LLM and the requested list length (200 genes; long and short input prompts). (B) Percentage of LLM-reported genes not contained in the cohort reference gene list (long and short input prompts), summarized across the same 100 attempts as in (A). In (A) and (B), each point represents one of 100 independent LLM attempts per cohort and model. Box plots show the median (center line), interquartile range (IQR; box), with whiskers extending to 1.5×IQR.

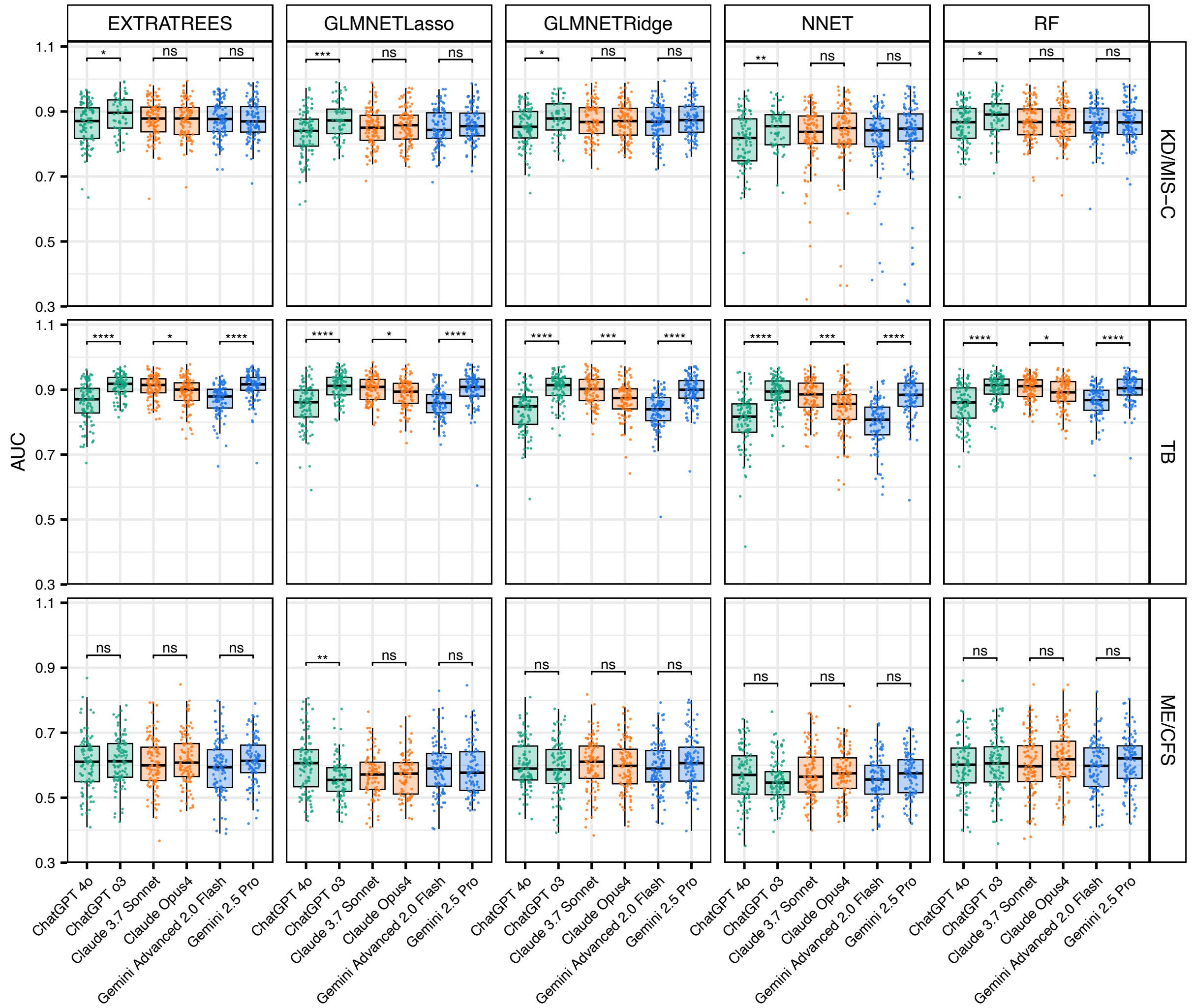

Supplementary Figure 2: Distribution of held-out test set ROC-AUCs across 100 random train-test splits, classifiers, and all LLM-driven gene-panel selection methods. Each point represents one independent LLM attempt per cohort and classifier. Box plots show the median (center line), interquartile range (IQR; box), with whiskers extending to 1.5×IQR. Brackets denote pairwise comparisons; ns indicates not significant, and asterisks indicate significance levels (**P* < 0.05, ***P* < 0.01, ****P* < 0.001, *****P* < 0.0001). Statistical significance was assessed using two-sided Welch's two-sample *t*-tests (up to *n* = 100 seeds per group) with Benjamini–Hochberg correction for multiple comparisons. Exact *P* and *q* values for each comparison are reported in Supplementary Table 1.

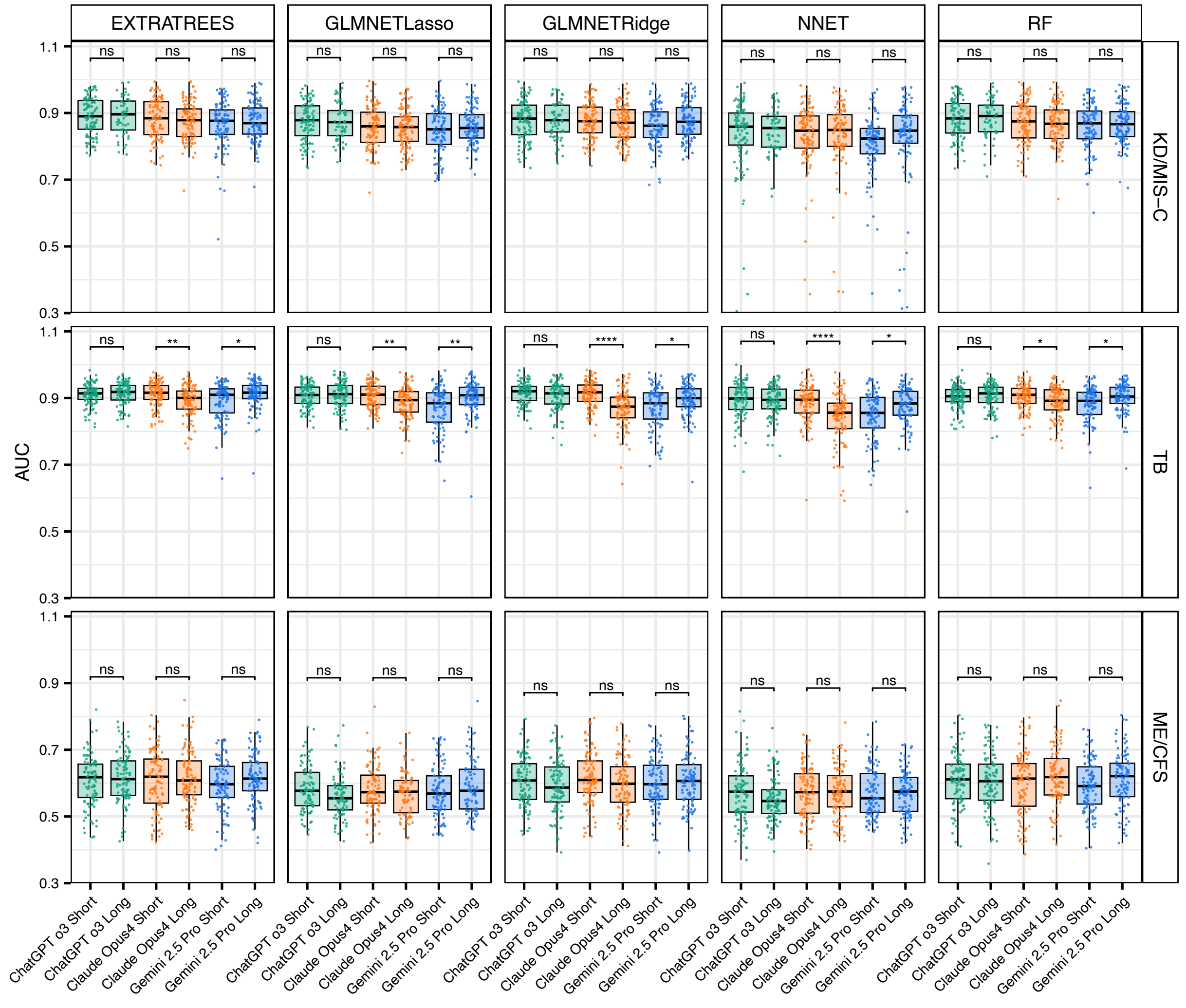

Supplementary Figure 3: Distribution of held-out test set ROC-AUCs across 100 random train-test splits, classifiers, and long- and short-prompted LLM-driven gene-panel selection methods. Each point represents one independent LLM attempt per cohort and classifier. Box plots show the median (center line), interquartile range (IQR; box), with whiskers extending to 1.5×IQR. Brackets denote pairwise comparisons; ns indicates not significant, and asterisks indicate significance levels (**P* < 0.05, ***P* < 0.01, ****P* < 0.001, *****P* < 0.0001). Statistical significance was assessed using two-sided Welch's two-sample t-tests (up to n = 100 seeds per group) with Benjamini-Hochberg correction for multiple comparisons. Exact P and q values for each comparison are reported in Supplementary Table 2.

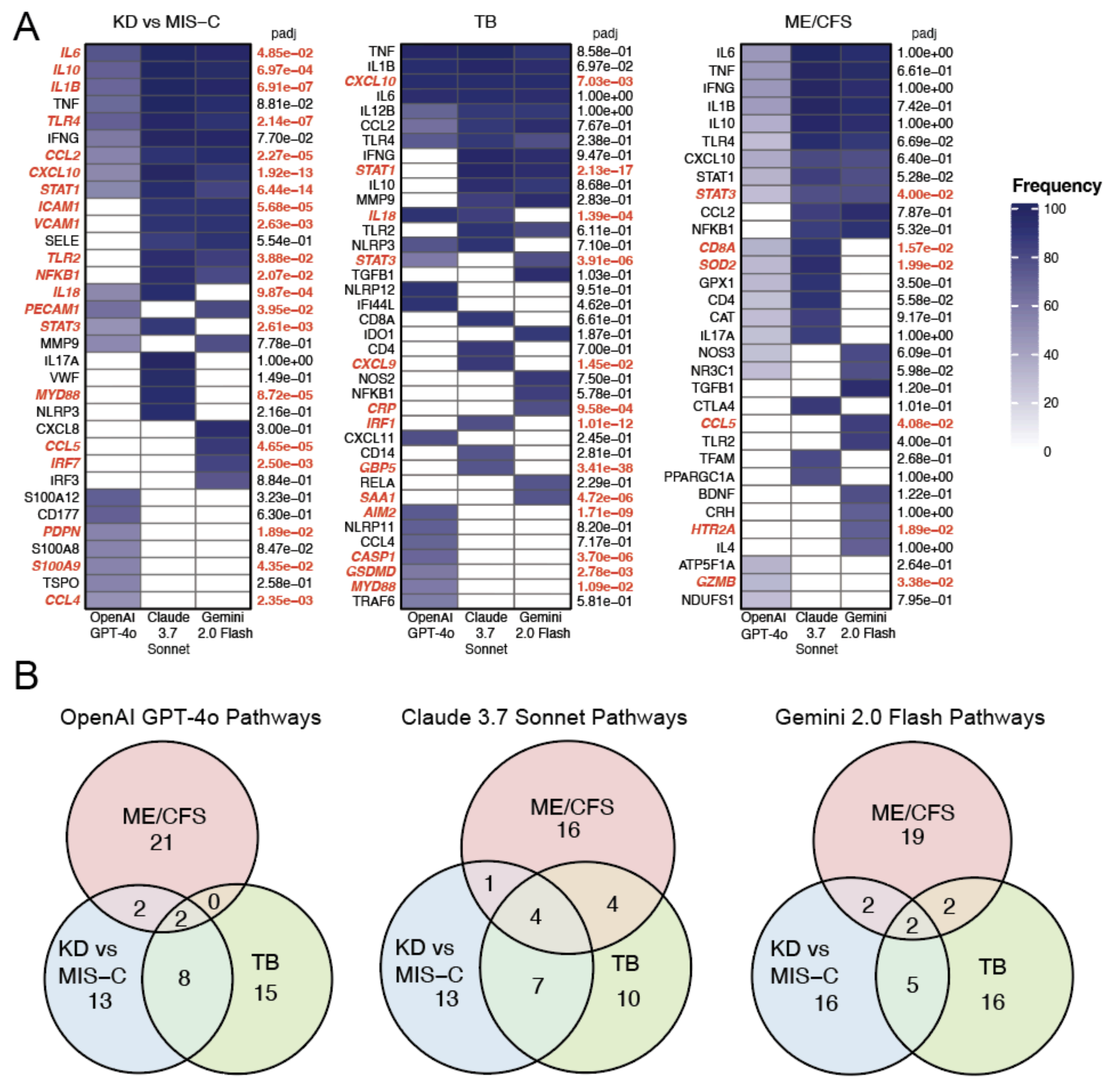

Supplementary Figure 4: LLM gene selection and biological correlation (OpenAI GPT-4o, Claude 3.7 Sonnet, Gemini 2.0 Flash). (A) Heatmaps summarizing the most frequently selected genes for each cohort across LLM-based feature selection and DESeq2-based differential expression. Heatmap color intensity indicates the gene selection frequency across 100 independent LLM attempts, from 0 (white) to 100 (dark blue). The DESeq2 *p*adj values (shown at right) are Benjamini–Hochberg-adjusted *P* values from differential gene expression analysis for each cohort comparison; gene symbols in red indicate *p*adj < 0.05. (B) Venn diagrams showing overlap among cohorts in the top 25 enriched pathways derived from each model's selected gene panel. Numbers indicate the count of pathways unique to each cohort or shared across cohorts.

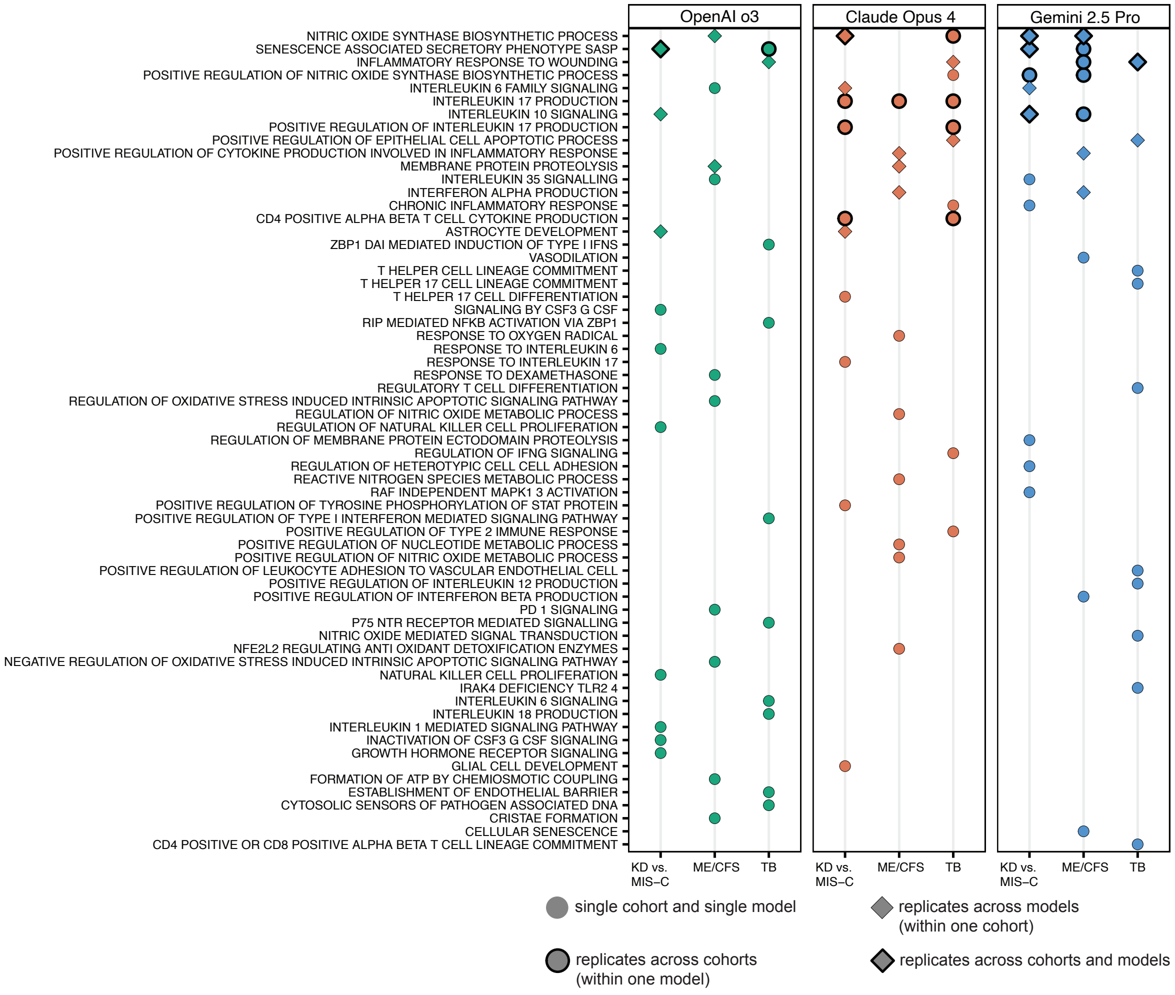

Supplementary Figure 5: Pathway enrichment across LLMs and cohorts. Points indicate that the pathway was identified as enriched for the specified cohort comparison (KD vs. MIS-C, ME/CFS, TB) by the indicated LLM (OpenAI o3, Claude Opus 4, Gemini 2.5 Pro). Marker shape encodes the breadth of replication: a circle indicates enrichment in one cohort by one model; a bordered circle indicates enrichment across multiple cohorts within one model; a diamond indicates enrichment across multiple models within one cohort; and a bordered diamond indicates enrichment across multiple cohorts and multiple models.

Supplementary Table 1: Pairwise statistical comparisons between LLM gene-panel selection methods (Supplementary Figure 2)									
**cohort**	**ML model**	**LLM 1**	**LLM 2**	**n group 1**	**n group 2**	**t statistic**	**degrees of freedom**	**raw P value**	**BH-adj P value**
KD/MIS-C	EXTRATREES	OpenAI GPT-4o	OpenAI o3	100	56	-2.807897545	128.2889	0.00577	0.01558
KD/MIS-C	GLMNETLasso	OpenAI GPT-4o	OpenAI o3	100	58	-4.005980541	138.5931	0.00010	0.00041
KD/MIS-C	GLMNETRidge	OpenAI GPT-4o	OpenAI o3	100	59	-2.688687702	130.4547	0.00811	0.01921
KD/MIS-C	NNET	OpenAI GPT-4o	OpenAI o3	100	59	-3.110294049	146.4265	0.00225	0.00674
KD/MIS-C	RF	OpenAI GPT-4o	OpenAI o3	100	58	-2.335875701	124.7578	0.02109	0.04520
ME/CFS	EXTRATREES	OpenAI GPT-4o	OpenAI o3	100	100	-0.878652447	194.3074	0.38067	0.53532
ME/CFS	GLMNETLasso	OpenAI GPT-4o	OpenAI o3	96	83	3.5224601	173.4188	0.00055	0.00176
ME/CFS	GLMNETRidge	OpenAI GPT-4o	OpenAI o3	100	100	1.118889703	196.7839	0.26455	0.39683
ME/CFS	NNET	OpenAI GPT-4o	OpenAI o3	100	100	1.502457045	185.5090	0.13468	0.23310
ME/CFS	RF	OpenAI GPT-4o	OpenAI o3	100	100	-0.453500096	197.9866	0.65069	0.83660
TB	EXTRATREES	OpenAI GPT-4o	OpenAI o3	100	100	-7.391586647	155.6931	8.3420E-12	6.2565E-11
TB	GLMNETLasso	OpenAI GPT-4o	OpenAI o3	100	100	-7.965266199	158.0187	3.0511E-13	2.7460E-12
TB	GLMNETRidge	OpenAI GPT-4o	OpenAI o3	100	100	-8.619609277	165.9840	5.0991E-15	7.6487E-14
TB	NNET	OpenAI GPT-4o	OpenAI o3	100	100	-8.801786537	156.3452	2.3705E-15	5.7551E-14
TB	RF	OpenAI GPT-4o	OpenAI o3	100	100	-6.980775005	163.9256	6.9347E-11	4.4580E-10
KD/MIS-C	EXTRATREES	Claude 3.7 Sonnet	Claude Opus 4	100	100	0.038677925	197.6394	0.96919	0.98022
KD/MIS-C	GLMNETLasso	Claude 3.7 Sonnet	Claude Opus 4	100	100	-0.649908093	197.9982	0.51650	0.70432
KD/MIS-C	GLMNETRidge	Claude 3.7 Sonnet	Claude Opus 4	100	100	0.203674547	197.9884	0.83882	0.96787
KD/MIS-C	NNET	Claude 3.7 Sonnet	Claude Opus 4	100	100	0.090609179	184.1512	0.92790	0.97998
KD/MIS-C	RF	Claude 3.7 Sonnet	Claude Opus 4	100	100	0.079860358	197.7651	0.93643	0.97998
ME/CFS	EXTRATREES	Claude 3.7 Sonnet	Claude Opus 4	100	100	-1.033524041	197.9991	0.30262	0.43929
ME/CFS	GLMNETLasso	Claude 3.7 Sonnet	Claude Opus 4	87	84	0.303313172	168.7906	0.76202	0.92679
ME/CFS	GLMNETRidge	Claude 3.7 Sonnet	Claude Opus 4	100	100	0.549837668	197.9193	0.58305	0.77168
ME/CFS	NNET	Claude 3.7 Sonnet	Claude Opus 4	100	100	0.099989164	196.5930	0.92045	0.97998
ME/CFS	RF	Claude 3.7 Sonnet	Claude Opus 4	100	100	-1.511590204	196.7483	0.13224	0.23310
TB	EXTRATREES	Claude 3.7 Sonnet	Claude Opus 4	100	100	2.71714939	188.1890	0.00720	0.01800
TB	GLMNETLasso	Claude 3.7 Sonnet	Claude Opus 4	100	100	2.366842329	193.9769	0.01893	0.04258
TB	GLMNETRidge	Claude 3.7 Sonnet	Claude Opus 4	100	100	3.843426768	188.1165	0.00017	0.00057
TB	NNET	Claude 3.7 Sonnet	Claude Opus 4	100	100	3.906655002	182.9107	0.00013	0.00049
TB	RF	Claude 3.7 Sonnet	Claude Opus 4	100	100	2.786089913	186.4389	0.00589	0.01558
KD/MIS-C	EXTRATREES	Gemini 2.0 Flash	Gemini 2.5 Pro	100	100	0.307097146	197.8698	0.75909	0.92679
KD/MIS-C	GLMNETLasso	Gemini 2.0 Flash	Gemini 2.5 Pro	100	100	-1.227418553	197.6695	0.22113	0.35538
KD/MIS-C	GLMNETRidge	Gemini 2.0 Flash	Gemini 2.5 Pro	100	100	-1.460717277	197.7944	0.14568	0.24280
KD/MIS-C	NNET	Gemini 2.0 Flash	Gemini 2.5 Pro	100	100	-0.228490936	186.4195	0.81952	0.96787
KD/MIS-C	RF	Gemini 2.0 Flash	Gemini 2.5 Pro	100	100	0.024818244	197.9945	0.98022	0.98022
ME/CFS	EXTRATREES	Gemini 2.0 Flash	Gemini 2.5 Pro	100	100	-1.874212032	194.4489	0.06240	0.12764
ME/CFS	GLMNETLasso	Gemini 2.0 Flash	Gemini 2.5 Pro	88	85	0.113253286	170.7716	0.90996	0.97998
ME/CFS	GLMNETRidge	Gemini 2.0 Flash	Gemini 2.5 Pro	100	100	-1.145816128	197.1511	0.25326	0.39299
ME/CFS	NNET	Gemini 2.0 Flash	Gemini 2.5 Pro	100	100	-1.525466095	197.9778	0.12874	0.23310
ME/CFS	RF	Gemini 2.0 Flash	Gemini 2.5 Pro	100	100	-1.511028439	197.9984	0.13238	0.23310
TB	EXTRATREES	Gemini 2.0 Flash	Gemini 2.5 Pro	100	100	-6.396538498	196.6025	1.1357E-9	5.6783E-9
TB	GLMNETLasso	Gemini 2.0 Flash	Gemini 2.5 Pro	99	100	-6.52886918	193.7915	5.6712E-10	3.1901E-9
TB	GLMNETRidge	Gemini 2.0 Flash	Gemini 2.5 Pro	100	100	-8.07768597	193.3262	6.9615E-14	7.8317E-13
TB	NNET	Gemini 2.0 Flash	Gemini 2.5 Pro	100	100	-8.614213398	191.9858	2.5578E-15	5.7551E-14
TB	RF	Gemini 2.0 Flash	Gemini 2.5 Pro	100	100	-5.823305856	193.2881	2.3708E-8	1.0669E-7

Supplementary Table 2: Pairwise statistical comparisons between short and long prompts for each LLM gene-panel selection method (Supplementary Figure 3)									
**cohort**	**ML model**	**LLM 1**	**LLM 2**	**n group 1**	**n group 2**	**t statistic**	**degrees of freedom**	**raw P value**	**BH-adj P value**
KD/MIS-C	EXTRATREES	OpenAI o3 (short prompt)	OpenAI o3 (long prompt)	100	56	-0.2303	115.3424	0.81823	0.89806
KD/MIS-C	EXTRATREES	Claude Opus 4 (short prompt)	Claude Opus 4 (long prompt)	100	100	0.8381	197.3371	0.40298	0.64764
KD/MIS-C	EXTRATREES	Gemini 2.5 Pro (short prompt)	Gemini 2.5 Pro (long prompt)	98	100	-0.9292	183.1503	0.35403	0.61680
KD/MIS-C	GLMNETLasso	OpenAI o3 (short prompt)	OpenAI o3 (long prompt)	100	58	0.0818	122.3357	0.93496	0.96872
KD/MIS-C	GLMNETLasso	Claude Opus 4 (short prompt)	Claude Opus 4 (long prompt)	100	100	0.5357	196.9688	0.59279	0.76007
KD/MIS-C	GLMNETLasso	Gemini 2.5 Pro (short prompt)	Gemini 2.5 Pro (long prompt)	97	100	-1.4277	184.4266	0.15508	0.36729
KD/MIS-C	GLMNETRidge	OpenAI o3 (short prompt)	OpenAI o3 (long prompt)	100	59	-0.0013	130.0318	0.99896	0.99896
KD/MIS-C	GLMNETRidge	Claude Opus 4 (short prompt)	Claude Opus 4 (long prompt)	100	100	0.9435	197.7261	0.34660	0.61680
KD/MIS-C	GLMNETRidge	Gemini 2.5 Pro (short prompt)	Gemini 2.5 Pro (long prompt)	98	100	-1.9367	193.2425	0.05424	0.18774
KD/MIS-C	NNET	OpenAI o3 (short prompt)	OpenAI o3 (long prompt)	100	59	-1.2258	154.9166	0.22214	0.45748
KD/MIS-C	NNET	Claude Opus 4 (short prompt)	Claude Opus 4 (long prompt)	100	100	0.5137	185.4004	0.60806	0.76007
KD/MIS-C	NNET	Gemini 2.5 Pro (short prompt)	Gemini 2.5 Pro (long prompt)	98	100	-0.8784	177.7564	0.38092	0.63487
KD/MIS-C	RF	OpenAI o3 (short prompt)	OpenAI o3 (long prompt)	100	58	-0.1820	118.7226	0.85592	0.91705
KD/MIS-C	RF	Claude Opus 4 (short prompt)	Claude Opus 4 (long prompt)	100	100	0.5584	196.7140	0.57720	0.76007
KD/MIS-C	RF	Gemini 2.5 Pro (short prompt)	Gemini 2.5 Pro (long prompt)	98	100	-0.9246	189.6187	0.35637	0.61680
TB	EXTRATREES	OpenAI o3 (short prompt)	OpenAI o3 (long prompt)	100	100	-0.4051	196.7651	0.68584	0.81218
TB	EXTRATREES	Claude Opus 4 (short prompt)	Claude Opus 4 (long prompt)	100	100	3.3575	187.3457	0.00095	0.00857
TB	EXTRATREES	Gemini 2.5 Pro (short prompt)	Gemini 2.5 Pro (long prompt)	97	100	-2.7913	186.2651	0.00580	0.02609
TB	GLMNETLasso	OpenAI o3 (short prompt)	OpenAI o3 (long prompt)	100	100	-0.7446	195.9067	0.45743	0.66402
TB	GLMNETLasso	Claude Opus 4 (short prompt)	Claude Opus 4 (long prompt)	100	100	3.4073	187.0911	0.00080	0.00857
TB	GLMNETLasso	Gemini 2.5 Pro (short prompt)	Gemini 2.5 Pro (long prompt)	97	100	-3.6124	181.5054	0.00039	0.00589
TB	GLMNETRidge	OpenAI o3 (short prompt)	OpenAI o3 (long prompt)	100	100	2.1646	187.5690	0.03168	0.11880
TB	GLMNETRidge	Claude Opus 4 (short prompt)	Claude Opus 4 (long prompt)	100	100	6.1400	173.1971	5.4775E-9	2.4649E-7
TB	GLMNETRidge	Gemini 2.5 Pro (short prompt)	Gemini 2.5 Pro (long prompt)	97	100	-3.0159	181.1380	0.00293	0.01884
TB	NNET	OpenAI o3 (short prompt)	OpenAI o3 (long prompt)	100	100	0.6301	196.2365	0.52934	0.72182
TB	NNET	Claude Opus 4 (short prompt)	Claude Opus 4 (long prompt)	100	100	4.7920	184.3708	3.3875E-6	7.6219E-5
TB	NNET	Gemini 2.5 Pro (short prompt)	Gemini 2.5 Pro (long prompt)	97	100	-3.0649	184.5914	0.00250	0.01878
TB	RF	OpenAI o3 (short prompt)	OpenAI o3 (long prompt)	100	100	-0.3070	195.4938	0.75914	0.85404
TB	RF	Claude Opus 4 (short prompt)	Claude Opus 4 (long prompt)	100	100	2.8013	188.3791	0.00562	0.02609
TB	RF	Gemini 2.5 Pro (short prompt)	Gemini 2.5 Pro (long prompt)	97	100	-2.8425	183.5020	0.00498	0.02609
ME/CFS	EXTRATREES	OpenAI o3 (short prompt)	OpenAI o3 (long prompt)	100	100	-0.6429	197.8805	0.52100	0.72182
ME/CFS	EXTRATREES	Claude Opus 4 (short prompt)	Claude Opus 4 (long prompt)	100	100	-0.8093	194.8130	0.41935	0.65013
ME/CFS	EXTRATREES	Gemini 2.5 Pro (short prompt)	Gemini 2.5 Pro (long prompt)	100	100	-1.7303	197.6951	0.08514	0.23945
ME/CFS	GLMNETLasso	OpenAI o3 (short prompt)	OpenAI o3 (long prompt)	94	83	2.2432	174.3219	0.02614	0.10695
ME/CFS	GLMNETLasso	Claude Opus 4 (short prompt)	Claude Opus 4 (long prompt)	92	84	1.3095	173.9892	0.19210	0.43223
ME/CFS	GLMNETLasso	Gemini 2.5 Pro (short prompt)	Gemini 2.5 Pro (long prompt)	89	85	-1.1736	169.8386	0.24218	0.47383
ME/CFS	GLMNETRidge	OpenAI o3 (short prompt)	OpenAI o3 (long prompt)	100	100	1.2207	196.5310	0.22366	0.45748
ME/CFS	GLMNETRidge	Claude Opus 4 (short prompt)	Claude Opus 4 (long prompt)	100	100	1.6626	197.8300	0.09797	0.25934
ME/CFS	GLMNETRidge	Gemini 2.5 Pro (short prompt)	Gemini 2.5 Pro (long prompt)	100	100	-0.7849	197.9846	0.43342	0.65013
ME/CFS	NNET	OpenAI o3 (short prompt)	OpenAI o3 (long prompt)	100	100	1.6225	184.8816	0.10641	0.26601
ME/CFS	NNET	Claude Opus 4 (short prompt)	Claude Opus 4 (long prompt)	100	100	-0.3471	197.8739	0.72892	0.84106
ME/CFS	NNET	Gemini 2.5 Pro (short prompt)	Gemini 2.5 Pro (long prompt)	100	100	0.0663	196.9781	0.94719	0.96872
ME/CFS	RF	OpenAI o3 (short prompt)	OpenAI o3 (long prompt)	100	100	0.4697	196.1066	0.63912	0.77731
ME/CFS	RF	Claude Opus 4 (short prompt)	Claude Opus 4 (long prompt)	100	100	-1.7593	196.2959	0.08009	0.23945
ME/CFS	RF	Gemini 2.5 Pro (short prompt)	Gemini 2.5 Pro (long prompt)	100	100	-1.8383	197.0940	0.06752	0.21703

Supplementary Table 3: Pairwise statistical comparisons for end-to-end LLM classifier performance (Figure 4)

cohort	model 1	model 2	n	t statistic	degrees of freedom	raw P value	BH-adj P value
KD vs MIS-C	claude_opus_4_informed_accuracy	EXTRATREES_accuracy	50	-22.6896	49	1.2035E-27	2.1663E-26
KD vs MIS-C	claude_opus_4_informed_accuracy	GLMNETLasso_accuracy	50	-19.7843	49	5.1791E-25	2.6635E-24
KD vs MIS-C	claude_opus_4_informed_accuracy	GLMNETRidge_accuracy	50	-22.0684	49	4.1650E-27	3.7485E-26
KD vs MIS-C	claude_opus_4_informed_accuracy	NNET_accuracy	50	-21.7916	49	7.3086E-27	4.3852E-26
KD vs MIS-C	claude_opus_4_informed_accuracy	RF_accuracy	50	-21.8967	49	5.8991E-27	4.2473E-26
KD vs MIS-C	claude_opus_4_naive_accuracy	EXTRATREES_accuracy	50	-17.4213	49	1.2306E-22	4.0274E-22
KD vs MIS-C	claude_opus_4_naive_accuracy	GLMNETLasso_accuracy	50	-18.8615	49	4.1212E-24	1.6485E-23
KD vs MIS-C	claude_opus_4_naive_accuracy	GLMNETRidge_accuracy	50	-18.6496	49	6.7092E-24	2.4153E-23
KD vs MIS-C	claude_opus_4_naive_accuracy	NNET_accuracy	50	-22.0977	49	3.9251E-27	3.7485E-26
KD vs MIS-C	claude_opus_4_naive_accuracy	RF_accuracy	50	-17.2542	49	1.8494E-22	5.5481E-22
KD vs MIS-C	claude_opus_4_naive_accuracy	claude_opus_4_informed_accuracy	50	1.6282	49	0.10989	0.14652
KD vs MIS-C	openai_o3_informed_accuracy	EXTRATREES_accuracy	50	-1.8293	49	0.07344	0.12018
KD vs MIS-C	openai_o3_informed_accuracy	GLMNETLasso_accuracy	50	-1.6801	49	0.09931	0.13751
KD vs MIS-C	openai_o3_informed_accuracy	GLMNETRidge_accuracy	50	-2.5258	49	0.01483	0.02850
KD vs MIS-C	openai_o3_informed_accuracy	NNET_accuracy	50	-2.8927	49	0.00568	0.01279
KD vs MIS-C	openai_o3_informed_accuracy	RF_accuracy	50	-1.1736	49	0.24623	0.27701
KD vs MIS-C	openai_o3_informed_accuracy	claude_opus_4_informed_accuracy	50	19.4998	49	9.7368E-25	4.3816E-24
KD vs MIS-C	openai_o3_informed_accuracy	claude_opus_4_naive_accuracy	50	14.4432	49	2.7532E-19	7.0797E-19
KD vs MIS-C	openai_o3_informed_accuracy	openai_o3_naive_accuracy	50	-0.1390	49	0.89003	0.89003
KD vs MIS-C	openai_o3_naive_accuracy	EXTRATREES_accuracy	50	-1.7685	49	0.08320	0.12480
KD vs MIS-C	openai_o3_naive_accuracy	GLMNETLasso_accuracy	50	-1.6951	49	0.09640	0.13751
KD vs MIS-C	openai_o3_naive_accuracy	GLMNETRidge_accuracy	50	-2.5201	49	0.01504	0.02850
KD vs MIS-C	openai_o3_naive_accuracy	NNET_accuracy	50	-3.0140	49	0.00407	0.00978
KD vs MIS-C	openai_o3_naive_accuracy	RF_accuracy	50	-1.2795	49	0.20676	0.24812
KD vs MIS-C	openai_o3_naive_accuracy	claude_opus_4_informed_accuracy	50	23.8431	49	1.2917E-28	4.6501E-27
KD vs MIS-C	openai_o3_naive_accuracy	claude_opus_4_naive_accuracy	50	15.3268	49	2.5171E-20	6.9705E-20
ME/CFS	claude_opus_4_informed_accuracy	EXTRATREES_accuracy	50	-3.8020	49	0.00040	0.00119
ME/CFS	claude_opus_4_informed_accuracy	GLMNETLasso_accuracy	50	-4.2540	49	9.4230E-05	0.00037
ME/CFS	claude_opus_4_informed_accuracy	GLMNETRidge_accuracy	50	-5.9474	49	2.8199E-07	4.2871E-06
ME/CFS	claude_opus_4_informed_accuracy	NNET_accuracy	50	-4.2289	49	0.00010	0.00037
ME/CFS	claude_opus_4_informed_accuracy	RF_accuracy	50	-3.8084	49	0.00039	0.00119
ME/CFS	claude_opus_4_naive_accuracy	EXTRATREES_accuracy	50	-5.6371	49	8.4385E-07	7.5946E-06
ME/CFS	claude_opus_4_naive_accuracy	GLMNETLasso_accuracy	50	-5.8806	49	3.5725E-07	4.2871E-06
ME/CFS	claude_opus_4_naive_accuracy	GLMNETRidge_accuracy	50	-7.3294	49	2.0523E-09	7.3881E-08
ME/CFS	claude_opus_4_naive_accuracy	NNET_accuracy	50	-5.0064	49	7.5690E-06	3.8926E-05
ME/CFS	claude_opus_4_naive_accuracy	RF_accuracy	50	-5.3746	49	2.1159E-06	1.5235E-05
ME/CFS	claude_opus_4_naive_accuracy	claude_opus_4_informed_accuracy	50	-1.4056	49	0.16615	0.23006
ME/CFS	openai_o3_informed_accuracy	EXTRATREES_accuracy	50	-1.8699	49	0.06748	0.10123
ME/CFS	openai_o3_informed_accuracy	GLMNETLasso_accuracy	50	-2.1690	49	0.03497	0.06625
ME/CFS	openai_o3_informed_accuracy	GLMNETRidge_accuracy	50	-4.3612	49	0.00007	0.00030
ME/CFS	openai_o3_informed_accuracy	NNET_accuracy	50	-2.0067	49	0.05031	0.08625
ME/CFS	openai_o3_informed_accuracy	RF_accuracy	50	-2.0307	49	0.04773	0.08592
ME/CFS	openai_o3_informed_accuracy	claude_opus_4_informed_accuracy	50	1.9290	49	0.05953	0.09317
ME/CFS	openai_o3_informed_accuracy	claude_opus_4_naive_accuracy	50	3.0630	49	0.00356	0.00870
ME/CFS	openai_o3_informed_accuracy	openai_o3_naive_accuracy	50	-1.7956	49	0.07873	0.11337
ME/CFS	openai_o3_naive_accuracy	EXTRATREES_accuracy	50	0.1451	49	0.88521	0.96569
ME/CFS	openai_o3_naive_accuracy	GLMNETLasso_accuracy	50	-0.1711	49	0.86486	0.96569
ME/CFS	openai_o3_naive_accuracy	GLMNETRidge_accuracy	50	-1.9805	49	0.05327	0.08717
ME/CFS	openai_o3_naive_accuracy	NNET_accuracy	50	0.0465	49	0.96309	0.99061
ME/CFS	openai_o3_naive_accuracy	RF_accuracy	50	-0.1981	49	0.84381	0.96569
ME/CFS	openai_o3_naive_accuracy	claude_opus_4_informed_accuracy	50	3.7397	49	0.00048	0.00134
ME/CFS	openai_o3_naive_accuracy	claude_opus_4_naive_accuracy	50	5.1546	49	4.5423E-06	2.7254E-05
TB	claude_opus_4_informed_accuracy	EXTRATREES_accuracy	50	-33.1393	49	3.2528E-35	5.8550E-34
TB	claude_opus_4_informed_accuracy	GLMNETLasso_accuracy	50	-31.0513	49	6.8460E-34	8.2153E-33
TB	claude_opus_4_informed_accuracy	GLMNETRidge_accuracy	50	-33.4203	49	2.1879E-35	5.8550E-34
TB	claude_opus_4_informed_accuracy	NNET_accuracy	50	-26.3778	49	1.2996E-30	5.8482E-30
TB	claude_opus_4_informed_accuracy	RF_accuracy	50	-30.6551	49	1.2460E-33	1.1214E-32
TB	claude_opus_4_naive_accuracy	EXTRATREES_accuracy	50	-28.9061	49	1.9118E-32	1.3765E-31
TB	claude_opus_4_naive_accuracy	GLMNETLasso_accuracy	50	-25.7097	49	4.2039E-30	1.6815E-29
TB	claude_opus_4_naive_accuracy	GLMNETRidge_accuracy	50	-27.7596	49	1.2419E-31	7.4513E-31
TB	claude_opus_4_naive_accuracy	NNET_accuracy	50	-23.7968	49	1.4102E-28	5.0766E-28
TB	claude_opus_4_naive_accuracy	RF_accuracy	50	-26.7330	49	7.0353E-31	3.6182E-30
TB	claude_opus_4_naive_accuracy	claude_opus_4_informed_accuracy	50	1.2390	49	0.22126	0.24892
TB	openai_o3_informed_accuracy	EXTRATREES_accuracy	50	-5.6022	49	9.5397E-07	2.2895E-06
TB	openai_o3_informed_accuracy	GLMNETLasso_accuracy	50	-4.6060	49	2.9437E-05	5.2986E-05
TB	openai_o3_informed_accuracy	GLMNETRidge_accuracy	50	-4.5386	49	3.6874E-05	6.3213E-05
TB	openai_o3_informed_accuracy	NNET_accuracy	50	-3.2011	49	0.00240	0.00346
TB	openai_o3_informed_accuracy	RF_accuracy	50	-5.1055	49	5.3827E-06	1.0765E-05
TB	openai_o3_informed_accuracy	claude_opus_4_informed_accuracy	50	16.5404	49	1.0887E-21	3.0147E-21
TB	openai_o3_informed_accuracy	claude_opus_4_naive_accuracy	50	15.0689	49	5.0122E-20	1.2889E-19
TB	openai_o3_informed_accuracy	openai_o3_naive_accuracy	50	-0.7694	49	0.44537	0.44537
TB	openai_o3_naive_accuracy	EXTRATREES_accuracy	50	-5.5207	49	1.2697E-06	2.8569E-06
TB	openai_o3_naive_accuracy	GLMNETLasso_accuracy	50	-4.8473	49	1.3034E-05	2.4696E-05
TB	openai_o3_naive_accuracy	GLMNETRidge_accuracy	50	-4.3769	49	6.3009E-05	0.00010
TB	openai_o3_naive_accuracy	NNET_accuracy	50	-3.0358	49	0.00384	0.00511
TB	openai_o3_naive_accuracy	RF_accuracy	50	-5.4731	49	1.5001E-06	3.1768E-06
TB	openai_o3_naive_accuracy	claude_opus_4_informed_accuracy	50	19.5789	49	8.1649E-25	2.6721E-24
TB	openai_o3_naive_accuracy	claude_opus_4_naive_accuracy	50	17.0589	49	2.9880E-22	8.9639E-22
