# Supplementary material for: Benchmarking large language models for cell-free RNA diagnostic biomarker discovery
Source: Nat Commun. 2026 Jun 11;17:7429. doi: 10.1038/s41467-026-74077-x (PMC13408832; doi:10.1038/s41467-026-74077-x)
Supplement: Supplementary file 2 — Reporting Summary [file 41467_2026_74077_MOESM2_ESM.pdf]

nature portfolio

Corresponding author(s): Iwijn De Vlaminck (id93@cornell.edu)

Last updated by author(s): Apr 27, 2026

Reporting Summary

Nature Portfolio wishes to improve the reproducibility of the work that we publish. This form provides structure for consistency and transparency in reporting. For further information on Nature Portfolio policies, see our Editorial Policies and the Editorial Policy Checklist.

Statistics

For all statistical analyses, confirm that the following items are present in the figure legend, table legend, main text, or Methods section.

n/a	Confirmed	
☐	☒	The exact sample size (*n*) for each experimental group/condition, given as a discrete number and unit of measurement
☐	☒	A statement on whether measurements were taken from distinct samples or whether the same sample was measured repeatedly
☐	☒	The statistical test(s) used AND whether they are one- or two-sided<br>*Only common tests should be described solely by name; describe more complex techniques in the Methods section.*
☐	☒	A description of all covariates tested
☐	☒	A description of any assumptions or corrections, such as tests of normality and adjustment for multiple comparisons
☐	☒	A full description of the statistical parameters including central tendency (e.g. means) or other basic estimates (e.g. regression coefficient) AND variation (e.g. standard deviation) or associated estimates of uncertainty (e.g. confidence intervals)
☐	☒	For null hypothesis testing, the test statistic (e.g. *F*, *t*, *r*) with confidence intervals, effect sizes, degrees of freedom and *P* value noted<br>*Give P values as exact values whenever suitable.*
☒	☐	For Bayesian analysis, information on the choice of priors and Markov chain Monte Carlo settings
☒	☐	For hierarchical and complex designs, identification of the appropriate level for tests and full reporting of outcomes
☒	☐	Estimates of effect sizes (e.g. Cohen's *d*, Pearson's *r*), indicating how they were calculated

Our web collection on statistics for biologists contains articles on many of the points above.

Software and code

Policy information about availability of computer code

Data collection	Large language model interactions were performed through the vendor web interfaces: OpenAI ChatGPT (models: o3, GPT-4o), Anthropic Claude (models: Opus 4, 3.7 Sonnet), and Google Gemini (models: Gemini 2.5 Pro, Gemini 2.0 Flash), accessed between 03/01/2025-08/01/2025.
Data analysis	Data analysis was performed in R v4.2.2 using the following packages: DESeq2 v1.38.0 (differential gene expression), GSVA v1.46.0 (single-sample gene-set enrichment), and caret v6.0.94 (machine learning, wrapping glmnet v4.1.8, randomForest v4.7.1.2, extraTrees v41.0.5, and nnet v7.3.19). Custom R scripts used for preprocessing, model evaluation, and figure generation are available at https://github.com/adb258/cfrna_ai_manuscript.

For manuscripts utilizing custom algorithms or software that are central to the research but not yet described in published literature, software must be made available to editors and reviewers. We strongly encourage code deposition in a community repository (e.g. GitHub). See the Nature Portfolio guidelines for submitting code & software for further information.

Data

Policy information about availability of data

All manuscripts must include a data availability statement. This statement should provide the following information, where applicable:

- Accession codes, unique identifiers, or web links for publicly available datasets
- A description of any restrictions on data availability
- For clinical datasets or third party data, please ensure that the statement adheres to our policy

The raw sequencing data and de-identified RNA-seq count matrices used in this study have been deposited in the NCBI Gene Expression Omnibus under accession codes GSE255555 (https://www.ncbi.nlm.nih.gov/geo/query/acc.cgi?acc=GSE255555), GSE255071 (https://www.ncbi.nlm.nih.gov/geo/query/acc.cgi?acc=GSE255071), GSE255073 (https://www.ncbi.nlm.nih.gov/geo/query/acc.cgi?acc=GSE255073), GSE255074 (https://www.ncbi.nlm.nih.gov/geo/query/acc.cgi?acc=GSE255074), and GSE293840 (https://www.ncbi.nlm.nih.gov/geo/query/acc.cgi?acc=GSE293840), with no access restrictions. The pairwise statistical comparisons underlying Figure 4B are provided in the Supplementary Information (Supplementary Data 3). The processed data used in this study, including the reference gene lists provided to LLMs, the LLM-generated gene panels for each model and cohort, the LLM-generated classifier predictions, and the numerical values underlying all main and supplementary figures, are available in the accompanying GitHub repository (https://github.com/adb258/cfrna_ai_manuscript), archived at Zenodo (DOI: 10.5281/zenodo.19656363).

Research involving human participants, their data, or biological material

Policy information about studies with human participants or human data. See also policy information about sex, gender (identity/presentation), and sexual orientation and race, ethnicity and racism.

Reporting on sex and gender	Sex was recorded in the original cohort metadata as male or female. Train–test splits were constructed with sex considered during partitioning to reduce imbalance across splits. Gender identity was not collected in the source datasets.
Reporting on race, ethnicity, or other socially relevant groupings	Race, ethnicity, and other socially relevant groupings were recorded in some cohorts but were not used to stratify train–test splits or for subgroup analyses, because doing so would create small cell sizes and unstable estimates. We therefore did not perform race/ethnicity-stratified performance analyses in this benchmark.
Population characteristics	This study analyzed secondary cfRNA datasets previously collected and published in the primary cohort papers. Beyond sex (male/female), additional participant-level demographic variables were not used for stratification or subgroup analyses in this benchmark. Detailed cohort characteristics are reported in the original publications.
Recruitment	No new participant recruitment was conducted for this study. All data were obtained from previously recruited cohorts described in the original publications.
Ethics oversight	The Cornell University IRB for Human Participants (2012010003), New York, NY, approved the protocols for this study. All samples and patient information were deidentified for analysis and shared with collaborating institutions.

Note that full information on the approval of the study protocol must also be provided in the manuscript.

Field-specific reporting

Please select the one below that is the best fit for your research. If you are not sure, read the appropriate sections before making your selection.

☒ Life sciences ☐ Behavioural & social sciences ☐ Ecological, evolutionary & environmental sciences

For a reference copy of the document with all sections, see nature.com/documents/nr-reporting-summary-flat.pdf

Life sciences study design

All studies must disclose on these points even when the disclosure is negative.

Sample size	No statistical method was used to predetermine sample size. Patient sample sizes were fixed by the previously published source cohort studies (KD: n = 115; MIS-C: n = 50; TB: n = 142; symptomatic controls: n = 109; ME/CFS: n = 93; sedentary controls: n = 75) and could not be modified in this secondary analysis. All available samples meeting the source studies' quality-control criteria were included. The number of replicate runs per condition (n = 100 LLM attempts for feature-selection benchmarking; n = 50 random train–test splits for end-to-end pipeline benchmarking) was chosen to balance computational cost against statistical power and was sufficient to resolve performance differences across models and conditions, as documented in Supplementary Data 1, 2, and 3.
Data exclusions	No additional participant-level exclusions were applied beyond those described in the original cohort publications. All samples available in the shared datasets were included in the benchmark.
Replication	All findings reported in this study are based on aggregate distributions across multiple independent runs rather than single-attempt results: feature-selection benchmarking used n = 100 independent LLM attempts per model, prompt, and cohort; end-to-end pipeline benchmarking used n = 50 independent random train–test splits per cohort. Pairwise statistical comparisons across these distributions are reported in Supplementary Data 1, 2, and 3 and were consistent across replicates within each comparison. No findings failed to replicate within this study; however, LLM outputs are non-deterministic and may differ if the same prompts are issued to future model versions or to different model checkpoints, which is a limitation discussed in the manuscript.

Randomization	This study did not involve experimental group assignment. For model evaluation, samples were randomly partitioned into training and held-out test sets using repeated random splits (70:30) under cohort-specific constraints.
Blinding	Blinding was not applicable in the conventional experimental sense because this was a retrospective secondary analysis. Test labels were withheld from the LLMs during end-to-end evaluation (the test set was provided without annotation), and performance was computed only after predictions were returned.

Reporting for specific materials, systems and methods

We require information from authors about some types of materials, experimental systems and methods used in many studies. Here, indicate whether each material, system or method listed is relevant to your study. If you are not sure if a list item applies to your research, read the appropriate section before selecting a response.

Materials & experimental systems

n/a	Involved in the study
☒	☐ Antibodies
☒	☐ Eukaryotic cell lines
☒	☐ Palaeontology and archaeology
☒	☐ Animals and other organisms
☒	☐ Clinical data
☒	☐ Dual use research of concern
☒	☐ Plants

Methods

n/a	Involved in the study
☒	☐ ChIP-seq
☒	☐ Flow cytometry
☒	☐ MRI-based neuroimaging

Plants

Seed stocks	*Report on the source of all seed stocks or other plant material used. If applicable, state the seed stock centre and catalogue number. If plant specimens were collected from the field, describe the collection location, date and sampling procedures.*
Novel plant genotypes	*Describe the methods by which all novel plant genotypes were produced. This includes those generated by transgenic approaches, gene editing, chemical/radiation-based mutagenesis and hybridization. For transgenic lines, describe the transformation method, the number of independent lines analyzed and the generation upon which experiments were performed. For gene-edited lines, describe the editor used, the endogenous sequence targeted for editing, the targeting guide RNA sequence (if applicable) and how the editor was applied.*
Authentication	*Describe any authentication procedures for each seed stock used or novel genotype generated. Describe any experiments used to assess the effect of a mutation and, where applicable, how potential secondary effects (e.g. second site T-DNA insertions, mosiacism, off-target gene editing) were examined.*
