# Supplementary material for: Benchmarking large language models for cell-free RNA diagnostic biomarker discovery
Source: Nat Commun. 2026 Jun 11;17:7429. doi: 10.1038/s41467-026-74077-x (PMC13408832; doi:10.1038/s41467-026-74077-x)
Supplement: Supplementary file 3 — Transparent Peer Review file [file 41467_2026_74077_MOESM3_ESM.pdf]

nature portfolio

Peer Review File

Benchmarking large language models for cell-free RNA diagnostic biomarker discovery

Corresponding Author: Dr Iwijn De Vlaminck

This file contains all reviewer reports in order by version, followed by all author rebuttals in order by version.

Version 0:

Reviewer comments:

Reviewer #1

(Remarks to the Author)
This manuscript presents a timely and relevant benchmark of six contemporary large language models (LLMs) for cfRNA-based diagnostic biomarker discovery. The authors evaluate the LLMs on two distinct tasks: (i) nominating diagnostic gene panels based on public knowledge and (ii) constructing an end-to-end machine learning classifier directly from raw count data. Using three clinical cohorts of varying diagnostic difficulty (KD vs. MIS-C, TB, and ME/CFS), the study finds that while LLM-nominated gene panels outperform random selection, they generally do not surpass traditional differential gene expression (DGE) analysis, with performance correlating with the extent of public knowledge on the disease. For the end-to-end task, only a subset of models could complete the workflow, with OpenAI's model performing comparably to a conventional pipeline on one cohort but not others. The work provides a valuable snapshot of the current capabilities and limitations of LLMs in this domain.

Pros (Major Strengths)
1) The study is well-designed, systematically comparing multiple state-of-the-art LLMs against standard bioinformatics baselines (DGE, random selection) across multiple real-world clinical datasets.
2) The selection of three distinct clinical cohorts, representing a spectrum of diagnostic difficulty and available literature, is a significant strength. It allows for a nuanced assessment of how LLM performance is modulated by the maturity of the existing knowledge base for a given disease.
3) The dual-task evaluation (gene-panel design vs. end-to-end modeling) provides a comprehensive view of LLM utility, distinguishing between their capacity for knowledge synthesis and code generation.
4) The authors are commendably transparent about negative results, such as the LLMs' failure to outperform DGE in two of three cohorts, the lack of performance gain from providing disease-specific context in the end-to-end task, and the frequent failure of some models to complete tasks.

Cons (Major Points for Revision)
The manuscript is promising but requires revision to address several key experimental and reporting limitations before it can be considered for publication.
1) The manuscript uses non-standard and potentially confusing names for the models, such as "o3", "Claude Opus 4", and "Gemini 2.5 Pro". These appear to be inconsistent with the official branding (e.g., GPT-4o, Claude 3 Opus, Gemini 1.5 Pro). Given the rapid evolution of these models, precise versioning is critical for reproducibility. The authors must clarify the exact model versions used (e.g., via API identifiers or release dates) and use consistent, official nomenclature throughout the text and figures.
2) For the end-to-end classifier evaluation (Fig. 4), the authors report only classification accuracy. In a clinical context, and especially with imbalanced datasets (e.g., KD n=115 vs. MIS-C n=50), accuracy is an insufficient and potentially misleading metric. The analysis should be expanded to include ROC-AUC, Precision-Recall AUC, sensitivity, and specificity to provide a more robust assessment of classifier performance.
3) The authors report that "none of the models produced code that ran unmodified outside the chat environment" and that it "required substantial manual integration, limiting reproducibility". This is a crucial finding that significantly tempers the claim of an "autonomous" or "end-to-end" workflow. This limitation is currently understated in a "User Experience" section and should be moved to the main results and discussed as a primary finding. The "end-to-end" task is better described as "LLM-assisted code generation," which still requires significant human expertise.
4) The conclusion that "performance did not differ appreciably between short and long prompts" is presented without deeper

investigation. This is a missed opportunity for insight into how LLMs utilize context. A more detailed analysis is warranted. For example, did the LLMs ignore the extra information in the long prompts, or was the information provided not specific enough to guide feature selection effectively? A qualitative analysis of the LLM-generated rationale could be very instructive here.
5) For the gene-panel task, the authors requested 200 genes from the LLMs but used only the top 100 for downstream modeling. The justification provided—that requesting 100 genes directly would lead to more unusable seeds due to prompt adherence issues —is not fully convincing and requires further explanation. Why would a request for a shorter list be harder for an LLM to fulfill? This workaround could introduce bias if the LLMs' ranking is not optimal.
6) The manuscript states that Gemini 2.5 Pro was dropped from the end-to-end task because it "timed out or refused execution". While this is a useful practical data point, the authors should provide more detail on the failure modes. Were different prompting strategies or data formatting approaches attempted to mitigate these errors? A more thorough description of the debugging process would add significant value for other researchers attempting similar workflows.

(Remarks on code availability)
The code repo link is not valid. The authors should carefully prepare their code and data and resubmit a valid and real code link.

Reviewer #2

(Remarks to the Author)
General:
This paper benchmarked some general LLM for the selection of a gene panel and a binary classification task based on cfRNA data. The setup of the benchmark is laudable, with research questions of increasing complexity, a good number of attempts (without information storage), two types of prompts differing in content and amount of information provided. Prompts are provided as suppl data, which is very useful. Some clear conclusions can be drawn from the benchmark: for relatively easy tasks, LLMs may perform on par with manually-created pipelines; for more complex tasks LLMs are lagging behind; prompt adherence is not optimal; the provision of (clinical) context unfortunately does not improve performance. The work could have more impact by not restricting the work to cfRNA analyses but extend it beyond other types of RNA-seq datasets.

Major comments:
-Abstract: the description of the different tasks, in particular for the end-to-end modeling task, is not clear enough.
-Abstract: conclusions may be phrased more clearly, see suggestions above
-It is unclear why the LLM-selected gene panel evaluation was done with 100 genes instead of 200. It is unclear why genes with transcript abundance >50 and absolute log2 fold change >1 were excluded.
-Captions to figures should be more descriptive. For example in Figure 1, it is not immediately clear that the dots represent 100 attempts and what boxes and whiskers reflect.
-Prompt adherence is overall not so good. This makes it difficult to control the analysis in the way desired by the user. This should be mentioned as a serious limitation of the current LLMs. The authors should also invest some time in how the prompt adherence can be improved (perhaps by including a second prompt) because there is not much difference between the current short and long prompt.
-Figure 2: only show lines, not colored surfaces; the surfaces do not help and are even blurring the results. Preferably, use not only color but also different types of dashes to discriminate between the different gene sets. Make explicit that these are the results for the test set and not the training set.
-The github link does not work. Code should be made available.
-The discussion section could be more elaborate. In particular, the authors should discuss how LLM performance can be improved and how they can be deployed as a reproducible instrument in research

Minor comments:
-Abstract. Line 25-26. They underperformed… wrong sentence construction

(Remarks on code availability)
The github page is not publicly available

Reviewer #3

(Remarks to the Author)
This manuscript explores the use of large language models (LLMs) to perform semi-supervised and unsupervised classification tasks for cell-free RNA (cfRNA) diagnostic biomarker discovery. The authors benchmark six LLMs (OpenAI, Anthropic, Google) against a standard cfRNA workflow based on differential gene expression (DGE) using DESeq2 followed by machine learning classifiers. They evaluate performance across three classification problems of increasing difficulty (KD vs. MIS-C, TB vs. symptomatic controls, and ME/CFS vs. controls), with the ME/CFS task being particularly challenging due to the absence of validated biomarkers.

The most surprising finding of the study is that while LLMs underperform standard workflows when tasked with biomarker discovery alone, they can achieve comparable performance to conventional pipelines when prompted to develop full end-to-end classification workflows. This represents a novel and timely contribution that will be of interest to the readership of Nature Communications, and I therefore recommend it for publication. Some aspects, however, would benefit from clarification or deeper analysis to strengthen the work and maximize its usefulness for researchers aiming to apply these approaches to their own datasets.

Main points:

• As noted in the abstract, a core advantage of LLMs is their ability to parse large datasets and generate executable code. In principle, having the models return the exact code used for classification would greatly improve interpretability and allow readers to understand which specific modeling decisions drive performance. However, the prompts explicitly prohibited LLMs from providing such code. Could the authors clarify the rationale for this choice?

• LLMs were explicitly instructed not to use information from previous papers from the authors analyzing the same datasets. However, it seems difficult to verify compliance, as LLMs may have internalized these papers during training, or information could leak from secondary sources. Is there any way to validate or quantify potential leakage? Even a small validation test on a completely unseen task (using a synthetic dataset or modified gene list) could help assess performance.

• Some of the prompts used in the study are very detailed. While this provides structure, it also makes it difficult to disentangle the effects of different prompt components. Can the authors discuss prompt length optimization for these tasks? A brief discussion on how prompt granularity influences LLM performance or other generalizable points would be useful. This could help future users understand how to balance reproducibility, performance, and interpretability when designing complex prompts for biomarker discovery.

Minor points:

• In the Methods section (p.16), the authors state that highly expressed genes with large log2 fold changes were excluded in the KD vs. MIS-C comparison. This seems counterintuitive. What is the rationale behind this?

• The manuscript discusses pathway overlaps between LLM-selected genes and DGE analyses. It would be useful to include a pathway enrichment plot and highlight potential differences between models and the conventional DGE analysis.

• The colors in figure 4 A for the boxes could be revised for clarity, as currently they suggest that there is something in common between the green boxes which might confuse the reader.

(Remarks on code availability)

Version 1:

Reviewer comments:

Reviewer #1

(Remarks to the Author)
Thank the authors for addressing most of my concerns.

(Remarks on code availability)

Reviewer #2

(Remarks to the Author)
The authors have addressed my concerns adequately.
The github repository is now available, but the documentation of the code is poor. The authors claim in their checklist that they provided installation instructions. Please add these to the github repository as well.

(Remarks on code availability)
The README is very limited. The results of the paper will be hard to reproduce

Reviewer #3

(Remarks to the Author)
I am satisfied with the authors’ responses and have no further comments.

(Remarks on code availability)

Version 2:

Reviewer comments:

Reviewer #2

(Remarks to the Author)
No remaining concerns

(Remarks on code availability)

Open Access This Peer Review File is licensed under a Creative Commons Attribution 4.0 International License, which permits use, sharing, adaptation, distribution and reproduction in any medium or format, as long as you give appropriate credit to the original author(s) and the source, provide a link to the Creative Commons license, and indicate if changes were made.
In cases where reviewers are anonymous, credit should be given to 'Anonymous Referee' and the source.
The images or other third party material in this Peer Review File are included in the article's Creative Commons license, unless indicated otherwise in a credit line to the material. If material is not included in the article's Creative Commons license and your intended use is not permitted by statutory regulation or exceeds the permitted use, you will need to obtain permission directly from the copyright holder.
To view a copy of this license, visit https://creativecommons.org/licenses/by/4.0/

Reviewers' comments:

(Author response in blue)

Reviewer #1 (Remarks to the Author):

This manuscript presents a timely and relevant benchmark of six contemporary large language models (LLMs) for cfRNA-based diagnostic biomarker discovery. The authors evaluate the LLMs on two distinct tasks: (i) nominating diagnostic gene panels based on public knowledge and (ii) constructing an end-to-end machine learning classifier directly from raw count data. Using three clinical cohorts of varying diagnostic difficulty (KD vs. MIS-C, TB, and ME/CFS), the study finds that while LLM-nominated gene panels outperform random selection, they generally do not surpass traditional differential gene expression (DGE) analysis, with performance correlating with the extent of public knowledge on the disease. For the end-to-end task, only a subset of models could complete the workflow, with OpenAI's model performing comparably to a conventional pipeline on one cohort but not others. The work provides a valuable snapshot of the current capabilities and limitations of LLMs in this domain.
We thank the reviewer for the appreciation of the timeliness and value of this work.

Pros (Major Strengths)
1) The study is well-designed, systematically comparing multiple state-of-the-art LLMs against standard bioinformatics baselines (DGE, random selection) across multiple real-world clinical datasets.
2) The selection of three distinct clinical cohorts, representing a spectrum of diagnostic difficulty and available literature, is a significant strength. It allows for a nuanced assessment of how LLM performance is modulated by the maturity of the existing knowledge base for a given disease.
3) The dual-task evaluation (gene-panel design vs. end-to-end modeling) provides a comprehensive view of LLM utility, distinguishing between their capacity for knowledge synthesis and code generation.
4) The authors are commendably transparent about negative results, such as the LLMs' failure to outperform DGE in two of three cohorts, the lack of performance gain from providing disease-specific context in the end-to-end task, and the frequent failure of some models to complete tasks.
We thank the reviewer for highlighting these major strengths of our study.

Cons (Major Points for Revision)
The manuscript is promising but requires revision to address several key experimental and reporting limitations before it can be considered for publication.
1) The manuscript uses non-standard and potentially confusing names for the models, such as "o3", "Claude Opus 4", and "Gemini 2.5 Pro". These appear to be inconsistent with the official branding (e.g., GPT-4o, Claude 3 Opus, Gemini 1.5 Pro). Given the rapid evolution of these models, precise versioning is critical for reproducibility. The authors must clarify the exact model versions used (e.g., via API identifiers or release dates) and use consistent, official nomenclature throughout the text and figures.

We now refer to all LLMs by their official branding names (OpenAI o3, OpenAI GPT-4o, Claude Opus 4, Claude 3.7 Sonnet, Gemini 2.5 Pro, Gemini 2.0 Flash) and write out the full name every time they are referenced in the text and figures.

2) For the end-to-end classifier evaluation (Fig. 4), the authors report only classification accuracy. In a clinical context, and especially with imbalanced datasets (e.g., KD n=115 vs. MIS-C n=50), accuracy is an insufficient and potentially misleading metric. The analysis should be expanded to include ROC-AUC, Precision-Recall AUC, sensitivity, and specificity to provide a more robust assessment of classifier performance.
We agree that accuracy alone can be misleading for imbalanced datasets and that ROC-AUC, PR-AUC, sensitivity, and specificity are often preferred. However, in the LLM-driven classifier construction and evaluation portion of our study, the LLM output consisted only of a discrete predicted class label for each subject ID, without any continuous decision score (e.g. predicted probability, margin, or logit). Because ROC-AUC and PR-AUC require a ranked score per sample to generate performance across thresholds, these metrics cannot be computed from these hard-label predictions alone. We highlight this limitation in the discussion section of the manuscript.

3) The authors report that "none of the models produced code that ran unmodified outside the chat environment" and that it "required substantial manual integration, limiting reproducibility". This is a crucial finding that significantly tempers the claim of an "autonomous" or "end-to-end" workflow. This limitation is currently understated in a "User Experience" section and should be moved to the main results and discussed as a primary finding. The "end-to-end" task is better described as "LLM-assisted code generation," which still requires significant human expertise.
We agree that this finding should be highlighted in the main "Results" section. We moved this limitation from the "User Experience" section to the end of the "LLMs autonomously build machine learning classifiers" section within "Results".

4) The conclusion that "performance did not differ appreciably between short and long prompts" is presented without deeper investigation. This is a missed opportunity for insight into how LLMs utilize context. A more detailed analysis is warranted. For example, did the LLMs ignore the extra information in the long prompts, or was the information provided not specific enough to guide feature selection effectively? A qualitative analysis of the LLM-generated rationale could be very instructive here.
We thank the reviewer for pointing this out. We added a reference to the relevant supplementary figures and files (Fig. S2 and S3; Files S6 and S7) in the statement that claims that there were no appreciable differences in prompt adherence and downstream predictive performance between the short and long prompts at the end of Results: "LLMs vary in their ability to adhere to prompt requirements". We agree that an in-depth analysis of LLM-generated rationale would be valuable. We are working on related qualitative analyses to better characterize the LLMs' approaches to biomarker discovery as part of a follow-up study.

5) For the gene-panel task, the authors requested 200 genes from the LLMs but used only the top 100 for downstream modeling. The justification provided—that requesting 100 genes directly would lead to more

unusable seeds due to prompt adherence issues —is not fully convincing and requires further explanation. Why would a request for a shorter list be harder for an LLM to fulfill? This workaround could introduce bias if the LLMs' ranking is not optimal.
We appreciate this comment and agree that this rationale should be clarified. Our decision to request 200 genes and retain the first 100 was not intended to improve panel quality, but to mitigate occasional prompt non-adherence in which LLMs returned fewer than the requested number of unique genes. We do not claim that requesting a shorter list is intrinsically harder for LLMs, rather, current LLMs just do not reliably satisfy specified list-length constraints. Under-generation becomes problematic when downstream preprocessing removes additional features (e.g., genes with near-zero variance or extreme sparsity across samples), potentially leaving an insufficient feature set and causing seed-to-seed variability and pipeline failures. Therefore, to preserve a fixed 100-gene panel design without iterative re-prompting and stitching together of LLM outputs (which would introduce variability in LLM interaction), we prompted the LLMs for a ranked list of 200 genes and applied a pre-specified cutoff by retaining only the first 100 genes prior to any downstream filtering. Importantly, we did not submit all 200 genes to downstream filtering and then select 100 that survived. QC and model-building steps were applied only after truncation, following the approach used in conventional ML. Thus, genes ranked >100 never enter the modeling workflow and cannot bias performance. We clarify this point and the associated cutoff rule in the revised manuscript (Methods: "Open-domain gene panel selection: performance benchmarking"; "Discussion").

6) The manuscript states that Gemini 2.5 Pro was dropped from the end-to-end task because it "timed out or refused execution". While this is a useful practical data point, the authors should provide more detail on the failure modes. Were different prompting strategies or data formatting approaches attempted to mitigate these errors? A more thorough description of the debugging process would add significant value for other researchers attempting similar workflows.
We agree that additional detail on failure modes would be valuable. We have expanded on the description of Gemini 2.5 Pro's failure modes in the revised manuscript (Results: "LLMs autonomously build machine learning classifiers"; Results: "User Experience"). In the end-to-end evaluation arm of the study, Gemini 2.5 Pro failed to complete the workflow reliably in two recurring ways: (1) it remained in an indefinite "thinking" state without producing the requested deliverables until the platform time limit was reached and (2) it terminated early with runtime/tool-level errors or refused execution. These behaviors were observed uniformly across cohorts and across the disease-informed and disease-naive prompt types. This prevented consistent completion of training and inference and caused Gemini 2.5 Pro to be excluded from the quantitative end-to-end comparison. Regarding mitigation attempts, our primary goal in this study was to benchmark models using a standardized and reproducible interaction protocol rather than to optimize each model through extensive prompt engineering or iterative debugging. We therefore limited mitigation to basic prompt re-issuance and consistent data packaging across models. We did not perform a systematic exploration of alternative formatting schemes or multi-stage prompting specifically tailored to Gemini, as this would introduce unequal optimization across models and substantially expand the scope of our study. We explicitly highlight this limitation.

Reviewer #1 (Remarks on code availability):

The code repo link is not valid. The authors should carefully prepare their code and data and resubmit a valid and real code link.

The code and dependent files are now available at: https://github.com/adb258/cfrna_ai_manuscript.

Reviewer #2 (Remarks to the Author):

General:
This paper benchmarked some general LLM for the selection of a gene panel and a binary classification task based on cfRNA data. The setup of the benchmark is laudable, with research questions of increasing complexity, a good number of attempts (without information storage), two types of prompts differing in content and amount of information provided. Prompts are provided as suppl data, which is very useful. Some clear conclusions can be drawn from the benchmark: for relatively easy tasks, LLMs may perform on par with manually-created pipelines; for more complex tasks LLMs are lagging behind; prompt adherence is not optimal; the provision of (clinical) context unfortunately does not improve performance. The work could have more impact by not restricting the work to cfRNA analyses but extend it beyond other types of RNA-seq datasets.
We thank the reviewer for the positive feedback on our study. We agree that incorporation of additional omics datasets would be interesting to explore as a future direction.

Major comments:
-Abstract: the description of the different tasks, in particular for the end-to-end modeling task, is not clear enough.
We agree that the description of tasks, specifically the end-to-end modeling, was insufficient, and have updated the abstract accordingly.

-Abstract: conclusions may be phrased more clearly, see suggestions above
We appreciate this comment and agree that the abstract was vague on conclusions. We have added clear takeaways, like those suggested above, to the end of the abstract.

-It is unclear why the LLM-selected gene panel evaluation was done with 100 genes instead of 200. It is unclear why genes with transcript abundance >50 and absolute log2 fold change >1 were excluded.
We appreciate this comment and agree that these decisions should be clarified. As we wrote in a response to a similar comment by reviewer 1, our decision to request 200 genes and retain the first 100 was to mitigate occasional prompt non-adherence in which LLMs returned fewer than the requested number of unique genes. The evaluated LLMs did not reliably satisfy specified list-length constraints. Under-generation becomes problematic when downstream preprocessing removes additional features (e.g., genes with near-zero variance or extreme sparsity across samples), potentially leaving an insufficient feature set and causing seed-to-seed variability and pipeline failures. Therefore, to preserve a fixed 100-gene panel design without iterative re-prompting and stitching together of LLM outputs (which would introduce variability in LLM interaction), we

prompted the LLMs for a ranked list of 200 genes and applied a pre-specified cutoff by retaining only the first 100 genes prior to any downstream filtering. Importantly, we did not submit all 200 genes to downstream filtering and then select 100 that survived. QC and model-building steps were applied only after truncation, identically to the classical panels. Thus, genes ranked >100 never enter the modeling workflow and cannot bias performance. We clarify this point in the discussion.

As for the DGE filtering criteria, this was a typo: we inadvertently described our DGE inclusion criteria as exclusion criteria. The description in Methods: "In-house machine learning pipeline development" is correct, and we have removed the redundant and incorrect description from Methods: "Open-domain gene panel selection: performance benchmarking", instead referring readers to the earlier "Methods" section. We also found that the rationale for the cohort-specific DGE filtering was insufficiently explained. To maintain methodological concordance with our previously published analyses of these cohorts, we applied the same pre-specified DGE filtering thresholds (DESeq2 baseMean and $|\log_2$ fold change$|$ cutoffs) used in our prior cohort-specific pipelines ([7,9,11]). These thresholds were adopted to facilitate direct comparability and were not optimized for the present benchmark. We have added this clarification to Methods: "In-house machine learning pipeline development".

-Captions to figures should be more descriptive. For example in Figure 1, it is not immediately clear that the dots represent 100 attempts and what boxes and whiskers reflect.
We have revised the captions for Figures 1–4 to clarify the plotted quantities and summary statistics. In particular, we now specify the unit of replication (100 independent LLM attempts in Fig. 1 and 100 random train–test splits/seeds where applicable), and we define the boxplot elements (median, interquartile range, and whiskers extending to 1.5×IQR). We also added brief descriptions of what the points/curves represent in each panel and clarified the meaning of significance annotations where present.

-Prompt adherence is overall not so good. This makes it difficult to control the analysis in the way desired by the user. This should be mentioned as a serious limitation of the current LLMs. The authors should also invest some time in how the prompt adherence can be improved (perhaps by including a second prompt) because there is not much difference between the current short and long prompt.
We agree that prompt adherence is a major limitation in using current LLMs as controllable tools in biomarker discovery, and we have revised the discussion to emphasize this more explicitly. Regarding improving prompt adherence, we agree this is an important direction, however, a systematic prompt-optimization study (including second-pass "self-check/correction" prompts and model-specific formatting) was beyond the scope of this benchmark and could confound comparisons by introducing unequal degrees of model-specific tuning. Our goal here was to evaluate performance under standardized, reproducible prompting protocols shared across LLMs. We now note these considerations in the manuscript and point to future work aimed at quantifying prompt-adherence failures and evaluating mitigation strategies, including prompt-optimization approaches and analyses of LLM decision-making in biomarker identification.

-Figure 2: only show lines, not colored surfaces; the surfaces do not help and are even blurring the results. Preferably, use not only color but also different types of dashes to discriminate between the different gene sets. Make explicit that these are the results for the test set and not the training set.
We have revised Fig. 2 to improve interpretability. We added distinct line types for each gene-panel selection method to improve discrimination. We also updated the x-axis label and figure caption to explicitly state that all ROC-AUC values are computed on the held-out test set across 100 random train-test splits.

-The github link does not work. Code should be made available.
The code and dependent files are now available at https://github.com/adb258/cfrna_ai_manuscript.

-The discussion section could be more elaborate. In particular, the authors should discuss how LLM performance can be improved and how they can be deployed as a reproducible instrument in research
In the revised "Discussion" we added a section outlining strategies to improve performance (hybrid workflows that couple LLM-driven literature synthesis with conventional statistical filtering) and recommendations for reproducible deployment including versioning prompts and model identifiers, standardizing data packaging and evaluation protocols, archiving intermediate outputs, and transparently reporting/quantifying failure and rerun behavior alongside performance metrics.

Minor comments:
-Abstract. Line 25-26. They underperformed… wrong sentence construction
We have corrected this typo.

Reviewer #2 (Remarks on code availability):

The github page is not publicly available
The code and dependent files are now available at https://github.com/adb258/cfrna_ai_manuscript.

Reviewer #3 (Remarks to the Author):

This manuscript explores the use of large language models (LLMs) to perform semi-supervised and unsupervised classification tasks for cell-free RNA (cfRNA) diagnostic biomarker discovery. The authors benchmark six LLMs (OpenAI, Anthropic, Google) against a standard cfRNA workflow based on differential gene expression (DGE) using DESeq2 followed by machine learning classifiers. They evaluate performance across three classification problems of increasing difficulty (KD vs. MIS-C, TB vs. symptomatic controls, and ME/CFS vs. controls), with the ME/CFS task being particularly challenging due to the absence of validated biomarkers.

The most surprising finding of the study is that while LLMs underperform standard workflows when tasked with biomarker discovery alone, they can achieve comparable performance to conventional pipelines when prompted to develop full end-to-end classification workflows. This represents a novel and timely contribution

that will be of interest to the readership of Nature Communications, and I therefore recommend it for publication. Some aspects, however, would benefit from clarification or deeper analysis to strengthen the work and maximize its usefulness for researchers aiming to apply these approaches to their own datasets.
We thank the reviewer for their thoughtful and constructive assessment of this work. We appreciate their positive evaluation of the novelty and potential impact of our study.

Main points:

• As noted in the abstract, a core advantage of LLMs is their ability to parse large datasets and generate executable code. In principle, having the models return the exact code used for classification would greatly improve interpretability and allow readers to understand which specific modeling decisions drive performance. However, the prompts explicitly prohibited LLMs from providing such code. Could the authors clarify the rationale for this choice?
We appreciate this point. In practice, however, we found that none of the evaluated models produced code that executed unmodified outside of their native chat environments. When prompted to provide extractable code for external execution, the output consistently failed to run and required extensive manual integration. Because this would introduce substantial human intervention (and model-specific debugging) and reduce comparability across models, our end-to-end evaluation was performed within each LLM's integrated coding interface and focused on autonomous completion of training and inference under a standardized protocol. We have clarified this limitation and its implications for reproducibility in the revised Results section.

• LLMs were explicitly instructed not to use information from previous papers from the authors analyzing the same datasets. However, it seems difficult to verify compliance, as LLMs may have internalized these papers during training, or information could leak from secondary sources. Is there any way to validate or quantify potential leakage? Even a small validation test on a completely unseen task (using a synthetic dataset or modified gene list) could help assess performance.
We thank the reviewer for this comment and agree that it is not possible to strictly verify that prior publications were not considered by pretrained LLMs, which may have internalized relevant content during training or could indirectly access it via secondary sources. However, to mitigate this risk, our prompts explicitly instructed models to exclude our prior analyses of the same datasets ("Plasma Cell-free RNA Signatures of Inflammatory Syndromes in Children" and "Circulating cell-free RNA in blood as a host response biomarker for detection of tuberculosis") while constraining gene selection to the provided set of genes measured in the cohort expression matrix. Additionally, in runs where responses included source links, we manually reviewed the cited sources and our prior publications did not surface among the links. We now clarify this limitation explicitly in the manuscript. We also note that the ME/CFS dataset had not been previously published by our group at the time of analysis, providing a cohort in which direct reuse of our group's prior analyses was not possible.

In addition, we find that the LLM results are not consistent with simple reproduction of our previously published signatures. If reuse of our prior panels dominated, performance would be expected to closely track DGE across both KD vs. MIS-C and TB cohorts. Instead, we observed discordant outcomes: LLM-nominated

panels underperformed the DGE baseline in KD vs. MIS-C, while they nearly matched DGE in TB. In addition, in the ME/CFS cohort, where no prior publication from our group existed, LLM panels achieved comparable performance to DGE analysis. Together, these observations suggest that leakage is unlikely to be the driver of the reported trends.

• Some of the prompts used in the study are very detailed. While this provides structure, it also makes it difficult to disentangle the effects of different prompt components. Can the authors discuss prompt length optimization for these tasks? A brief discussion on how prompt granularity influences LLM performance or other generalizable points would be useful. This could help future users understand how to balance reproducibility, performance, and interpretability when designing complex prompts for biomarker discovery.
We thank the reviewer for this suggestion and agree that prompt granularity can influence performance and should be evaluated. We explicitly addressed this in our study design by using paired "short" and "long" prompts for each cohort to test whether additional structure and biological guidance improved prompt adherence or downstream predictive performance. As shown in Supplementary Fig. S1 ("Prompt adherence comparison by prompt length") and Supplementary Fig. S3 ("Distribution of held-out test set ROC-AUCs across 100 random train-test splits, classifiers, and "long"- and "short"-prompted LLM-driven gene-panel selection methods"), increasing prompt detail did not produce a consistent improvement in adherence metrics and did not yield a consistent increase in test-set ROC–AUC across cohorts/classifiers. More granular evaluation of individual prompt components could help identify which elements improve adherence and performance, and we consider this an important direction for future work.

Minor points:

• In the Methods section (p.16), the authors state that highly expressed genes with large log2 fold changes were excluded in the KD vs. MIS-C comparison. This seems counterintuitive. What is the rationale behind this?
We thank the reviewer for catching this. This was a typo, which we now fixed. We described our cohort-specific DGE inclusion criteria as exclusion criteria.

• The manuscript discusses pathway overlaps between LLM-selected genes and DGE analyses. It would be useful to include a pathway enrichment plot and highlight potential differences between models and the conventional DGE analysis.
We have added a supplementary figure (Fig. S5) that shows pathway enrichment across LLMs and cohorts. Pathway enrichment is also summarized in Fig. 3B, which reports overlap among the top enriched pathways (top 25) derived from each model's selected gene panel and enables comparison across cohorts and models.

• The colors in figure 4 A for the boxes could be revised for clarity, as currently they suggest that there is something in common between the green boxes which might confuse the reader.
We have changed the colors in the flowcharts (in both Fig. 1 and Fig. 4) accordingly.

Reviewers' comments:

(Author response in blue)

Reviewer #1 (Remarks to the Author):

Thank the authors for addressing most of my concerns.
We thank the reviewer for their evaluation of the revised manuscript and are glad to hear that our revisions addressed their concerns.

Reviewer #2 (Remarks to the Author):

The authors have addressed my concerns adequately.
The github repository is now available, but the documentation of the code is poor. The authors claim in their checklist that they provided installation instructions. Please add these to the github repository as well.

Reviewer #2 (Remarks on code availability):

The README is very limited. The results of the paper will be hard to reproduce
We thank Reviewer 2 for this feedback. We have substantially expanded the GitHub repository README (https://github.com/adb258/cfrna_ai_manuscript) to include: (1) full system requirements, including R version, all package dependencies with installation commands, and operating systems tested; (2) step-by-step installation instructions with estimated install time; (3) a demo section with instructions for running the analysis, expected output, and expected run time; and (4) detailed instructions for reproducing all manuscript results as well as guidance for applying the framework to new datasets. We have also added an MIT license to the repository and updated the Software Submission Checklist accordingly.

Reviewer #3 (Remarks to the Author):

I am satisfied with the authors' responses and have no further comments.
We thank the reviewer for their positive assessment of the revisions and are grateful for their feedback throughout the review process.
